# An Uncertainty-Robust Decision Framework Based on Fractional Einstein Aggregation for Prioritizing Renewable Power Generation Technologies with Unknown Weights

**DOI:** 10.3390/e28101129

**Published:** 2026-10-14

**Authors:** Majed Albaity, Muhammad Rahim

**Affiliations:** 1 Department of Mathematics, Faculty of Science, King Abdulaziz University, P.O. Box 80348, Jeddah 22254, Saudi Arabia; 2 Department of Mathematics and Statistics, University of Swat, Swat 19200, Pakistan

**Keywords:** renewable power generation, fractional orthopair fuzzy sets, Einstein aggregation operators, decision making, TOPSIS method

## Abstract

Renewable power generation plays a central role in the transition toward sustainable, low-carbon energy systems. However, selecting an appropriate technology remains challenging because technical, economic, environmental, and operational criteria may conflict, while expert judgments may involve uncertainty and diversity. To address these challenges, this study proposes a fractional fuzzy multi-criteria group decision-making framework for evaluating and ranking renewable power generation technologies under uncertain expert assessments. A new family of aggregation operators is developed by incorporating fractional fuzzy information into the Einstein operational framework, providing a mechanism for representing and aggregating uncertain assessments. An entropy-based weighting mechanism is further employed to determine criterion weights from the available decision information. The proposed components are integrated into a unified decision procedure that combines expert weighting, fractional fuzzy aggregation, criterion weighting, and distance-based TOPSIS assessment within a common computational framework. The numerical case study considers technical, economic, environmental, and operational criteria for renewable power generation technology selection. A sensitivity analysis examines the influence of the fractional parameters on the resulting rankings, while a comparative analysis with existing decision-making approaches is conducted to examine the consistency of the obtained results. The findings demonstrate the application of the proposed framework under the specified assessment conditions and illustrate the role of fractional fuzzy information and Einstein-based aggregation in the decision process. The study provides an integrated approach for incorporating uncertain expert information into renewable energy technology selection.

## 1. Introduction

In modern energy systems, renewable energy offers several important benefits [1,2]. For instance, it reduces reliance on imported fuels [3], enhances energy security [4], and can contribute to greater long-term electricity price stability [5,6]. Renewable energy resources also help reduce greenhouse gas emissions and air pollution, thereby contributing to improved environmental quality and public health [7,8]. Given the limitations of conventional energy sources, the global energy sector is undergoing a significant transition as many countries transition from fossil fuels to renewable energy sources. In recent years, solar, wind, hydro, biomass, and geothermal technologies have experienced rapid growth in deployment, driven by declining costs, improving efficiency, supportive policies, increased efficiency, and supportive policies. At the end of 2024, global renewable power capacity exceeded 4400 GW, with solar and wind accounting for most new capacity additions. The rapid expansion demonstrates that renewable energy sources have become increasingly important to modern energy systems. Despite this development, many regions still face insufficient renewable energy capacity to support growing electricity demand and climate targets.

Renewable energy planning is a complex process that requires consideration of technical, economic, environmental, and operational factors. Uncertainty and incomplete information further complicate the process. The development and deployment of renewable energy technologies are influenced by geographical location [9], environmental conditions [10], technological characteristics [11], policy frameworks, and market fluctuations. These factors complicate the prioritization of renewable power generation technologies, particularly when multiple and conflicting criteria must be considered simultaneously. Conventional decision-making approaches may have limitations in handling such uncertainties and in accommodating diverse judgments from multiple experts. To address these challenges, multi-criteria group decision-making (MCDM) methods have been widely applied [12,13,14,15]. MCDM provides a systematic framework for evaluating alternatives across multiple, often conflicting, criteria. However, most conventional MGDM approaches assume precise numerical data and a single decision-maker [16,17,18,19]. These approaches are inherently limited: they cannot accommodate group decision-making, nor can they operate effectively under uncertainty. To overcome this limitation, MCGDM techniques have been developed. MCGDM allows multiple experts to contribute simultaneously, yielding more reliable and representative outcomes. It has been applied across diverse domains, including technology assessment, site selection, energy policy analysis, and investment planning [20,21,22]. Despite their practicality, however, traditional MCGDM models often fail to capture uncertainty, hesitation, or non-linear interactions among criteria. Many also struggle when criteria weights are unknown or only partially known—a common and persistent challenge in real-world renewable energy planning.

Fuzzy set (FS) theory [23] provides a better way to handle information that is imprecise, vague, or expressed in linguistic terms, by assigning each element a degree of membership rather than a binary membership value. The underlying principle differs fundamentally from classical set theory, where an element either belongs to a set or does not. In contrast, a fuzzy set permits partial membership, represented by a value in the interval [0,1], which makes it well suited to problems involving gradual, imprecise, or poorly defined boundaries. Since ambiguity and subjectivity frequently arise in decision-making, FSs have been widely applied across engineering [24], economics [25,26], medical decision-making [27,28], and multi-criteria decision-making [29,30]. Classical FS theory has been extended through several generalizations, to provide richer representations of uncertainty and hesitation encountered in real-world decision-making. Since classical FSs capture only membership degrees (MDs) and does not explicitly represent non-membership degrees (NMDs). To address these shortcomings, intuitionistic fuzzy sets (IFSs) [31] were introduced. IFSs allow MDs and NMDs to be specified simultaneously, providing a more comprehensive representation of uncertain information. Different scholars have applied IFSs in various fields, including decision analysis, pattern recognition, and risk assessment [32,33]. The limitations of IFSs are addressed through several extensions including Pythagorean fuzzy sets (PFSs) [34,35], Fermatean fuzzy sets (FFSs) [36], *q*-rung orthopair fuzzy sets (*q*-ROFSs) [37], and (*p*, *q*)-quasirung orthopair fuzzy sets ((*p*, *q*)-QOFSs) [38,39].

Classical orthopair fuzzy frameworks are generally characterized by integer-valued power parameters. Let *γ*, *λ* ∈ [0,1] denote the MD and NMD, respectively. An orthopair (𝒰,𝒱) is admissible under the *q*-ROFS if *γ^q^* + *λ^q^* ≤ 1, *q* ≥ 1. Several well-known fuzzy frameworks arise as special cases of this constraint. For an IFS, *q* = 1, and hence *γ* + *λ* ≤ 1. For the PFS, *q* = 2, giving *γ*^2^ + *λ*^2^ ≤ 1, whereas a FFS corresponds to *q* = 3, with *γ*^3^ + *λ*^3^ ≤ 1. More generally, a *q*-ROFS employs an integer-valued *q* satisfying *q* ≥ 1. Consequently, the admissible domains generated by these frameworks form a discrete sequence 𝒟_1_ ⊆ 𝒟_2_ ⊆ 𝒟_3_, where 𝒟*_q_* = {(*γ*, *λ*) ∈ [0,1]^2^:*γ^q^* + *λ^q^* ≤ 1}. The inclusion 𝒟*_q_*_1_ ⊆ 𝒟*_q_*_2_ holds whenever *q*1 ≤ *q*2, because *γ*, *λ* ∈ [0,1] implies *γ^q^*^2^ ≤ *λ^q^*^1^ and *λ^q^*^2^ ≤ *λ^q^*^1^. However, the discrete nature of the integer-valued power parameter can restrict the representation of information that lies between two consecutive frameworks. For example, consider the orthopair (*γ*,*λ*) = (0.80,0.60). Under the IFS constraint, *γ* + *λ* = 0.80 + 0.60 = 1.40 > 1, so (0.80,0.60) ∉ 𝒟_1_. Under the PFS constraint, *γ*^2^ + *λ*^2^ = 0.80^2^ + 0.60^2^ = 0.64 + 0.36 = 1, and therefore (0.80,0.60) ∈ 𝒟_2_.

Although the information is admissible under the PFS framework, the integer-valued sequence *q* = 1,2,3,… does not provide a parameter between 1 and 2 for representing the gradual transition from the IFS domain to the PFS domain. A fractional power parameter can overcome this limitation by allowing *q* ∈ ℝ (where ℝ denote the set of real numbers), *q* ≥ 1. For the same orthopair (0.80,0.60), consider *q* = 1.50. Then 0.80^1.50^ + 0.60^1.5^ ≈ 0.7155 + 0.4648 = 1.1803 > 1, therefore, the orthopair is still inadmissible. At *q* = 1.8, 0.80^1.80^ + 0.60^1.80^ ≈ 0.6690 + 0.3987 = 1.0677 > 1, whereas at *q* = 2, 0.80^2^ + 0.60^2^ ≤ 1. Thus, a suitable fractional value *q* ∈ (1, *∞*) can identify the precise boundary at which a given orthopair becomes admissible. More generally, for a fixed orthopair (*γ*, *λ*) ∈ [0,1]^2^, define *F*(*q*) = *γ^q^* + *λ^q^*. The function *F*(*q*) is continuous and strictly decreasing in *q*, since *F′*(*q*) = *γ^q^ ln*(*γ*) + *λ^q^ ln*(*λ*) < 0. Therefore, if *F*(1) > 1 and *F*(2) ≤ 1, the intermediate value theorem guarantees the existence of a unique q* ∈ (1,2] such that $\gamma^{q^{*}}+\lambda^{q^{*}}=1$. Consequently, *γ^q^* + *λ^q^* ≤ 1 for all *q* ≥ q*. The same reasoning applies to any two consecutive integer powers *k* and *k* + 1. If *γ^k^* + *λ^k^* > 1 and *γ^k^*^+1^ + *λ^k^*^+1^ ≤ 1, then there exists a unique q* ∈ (*k*, *k* + 1] satisfying $\gamma^{q^{*}}+\lambda^{q^{*}}=1$. Hence, restricting the power parameter to integers produces a sequence of discrete admissible domains, whereas allowing real-valued powers generates the continuous family 𝒟*_α_* = {(*γ*, *λ*) ∈ [0,1]^2^:*γ^α^* + *λ^α^* ≤ 1}, *α* ≥ 1. Fractional orthopair fuzzy sets (FOFSs) [40] exploit this continuous parameterization to represent orthopair information that may not be adequately characterized by the conventional integer-powered frameworks. Selecting *α* ∈ (*k*, *k* + 1) provides an intermediate admissible domain satisfying 𝒟*_k_* ⊆ 𝒟*_α_* ⊆ 𝒟*_k_*_+1_. Thus, fractional extension does not merely introduce another fuzzy-set category; it provides a continuous mechanism for controlling the admissible region and enables a more granular representation of uncertain information.

Aggregation operators (AOs) play an important role in multi-criteria and group decision-making. Given the importance of AOs in combining information from multiple criteria and experts, several types of AOs have been developed in the decision-making literature to address diverse aggregation requirements. The weighted averaging operator [41] aggregates the input values according to their assigned weights, thereby reflecting the relative importance of the criteria or experts. In contrast, the weighted geometric operator [42] combines the inputs multiplicatively and provides an alternative mechanism for incorporating the relative importance of the inputs. The ordered weighted averaging operator [43] first arranges the input values according to their magnitudes and then assigns weights to their ordered positions, allowing the aggregation process to account for the relative ordering of the inputs. Hybrid AOs [44] combine the weighting mechanism of weighted averaging with the ordering mechanism of ordered weighted averaging, thereby considering both the importance and ordered positions of the inputs. The Bonferroni mean [45,46] and Heronian mean operators [47] incorporate interrelationships among the aggregated inputs and are therefore useful when interactions among criteria need to be considered [48]. The Muirhead mean provides a generalized parameterized structure for capturing interactions among multiple inputs [49]. Power AOs employ support-based mechanisms to regulate the influence of dissimilar input values and can therefore improve the robustness of aggregation [50]. In addition, AOs based on alternative t-norms and t-conorms, such as Einstein, Hamacher, and Dombi operators, provide flexible operational mechanisms for combining uncertain information [51,52,53]. Such operators have been extensively incorporated into fuzzy, intuitionistic fuzzy, and orthopair fuzzy environments to facilitate the aggregation of membership and non-membership information in multi-criteria decision-making problems [54,55].

Einstein AOs constitute an important class of aggregation methods based on Einstein t-norm and t-conorm operations, rather than conventional algebraic product and sum. The corresponding operational laws provide a continuous mechanism for combining fuzzy information and offer greater flexibility in modeling the interaction between membership and non-membership information. Their formulation is useful when expert assessments contain simultaneous degrees of confidence and hesitation, as the underlying operations allow the two components to be incorporated within a unified aggregation process. Compared with conventional aggregation mechanisms, Einstein operations provide an alternative algebraic structure that expands the range of possible aggregation behaviors and enables more flexible control over the resulting aggregated information. Owing to these characteristics, Einstein-based operators have been extended to various fuzzy environments, including intuitionistic [56], Pythagorean [57], and *q*-rung orthopair [58], where they provide an effective mechanism for aggregating uncertain assessments in multi-criteria group decision-making problems [59].

Keeping in mind the advantages of Einstein-based aggregation operators, we note that there is no study on Einstein aggregation operators under fractional orthopair fuzzy (FOF) sets. In this paper, we present a set of AOs to integrate expert assessments. It is also important to determine the importance of criteria. Subjective weighting methods are prone to bias, whereas entirely objective methods may disregard expert knowledge. Entropy-based weighting schemes address these issues by deriving weights from data while preserving the informational content of expert evaluations. Based on these observations, this study develops a new MCGDM framework for prioritizing renewable power generation technologies where criteria weights are unknown. The framework integrates FOFSs with Einstein t-norms to develop hybrid AOs. Criteria importance is determined using an entropy-based method, and alternatives are ranked using a TOPSIS-based procedure. The framework addresses uncertainty, expert hesitation, and conflicting criteria in a unified manner. Its performance is illustrated through a numerical example, and sensitivity analysis is conducted to test robustness.

This study aims to fill the above-mentioned gap by developing a comprehensive MCGDM framework for renewable power generation technology selection. The main contributions are:

(i) A flexible group decision framework based on FOFSs to represent uncertainty, hesitation, and varying confidence levels.
(ii) Formulation of Einstein-based AOs to combine expert evaluations reliably and account for nonlinear interactions.
(iii) An entropy-driven weighting method combined with a TOPSIS ranking procedure to determine criteria importance and prioritize technologies objectively.
(iv) Validation of the framework through a numerical example with multiple renewable technologies, covering technical, economic, environmental, and operational aspects.
(v) Sensitivity analysis to assess robustness and stability under varying uncertainty conditions.
(vi) Comparison with existing methods to demonstrate the advantages of the proposed approach.

This paper is organized as follows. Section 2 provides the fundamental concepts related to the proposed framework. In Section 3, new aggregation operators are proposed to integrate expert assessments. Section 4 presents a TOPSIS-based MCGDM approach for decision-making problems with fractional orthopair fuzzy information. In Section 5, the approach is applied to a numerical example, and sensitivity analysis is conducted to evaluate the robustness of the proposed framework. Section 6 concludes the paper and summarizes the proposed framework.

## 2. Preliminaries

This section presents the concepts of FOFSs and the Einstein t-norm and t-conorm which form the basis of the proposed framework.

**Definition** **1** ([40])**.** *Consider G* = {*g*_1_, *g*_2_, …, *g_n_*} *be a universal set. A fractional orthopair fuzzy set Z over G as defined as:*

*Z* = {*g*, 〈*γ_Z_*(*g*), *λ_Z_*(*g*)〉 | *g* ∈ *G*)} (1)

*In Equation (1), γ_Z_*(*g*), *λ_Z_*(*g*) ∈ [0,1] *denote the membership degree (MD) and non-membership degree (NMD), respectively, satisfying* $0\leq {\left(\gamma_{Z}\left(g\right)\right)}^{p/q}+{\left(\lambda_{Z}\left(g\right)\right)}^{p/q}\leq 1$ *for p*, *q* ≥ 1 *and p* ≥ *q. The corresponding hesitancy degree is given by:* $\pi_{Z}\left(g\right)={\left(1-{\left(\gamma_{Z}\left(g\right)\right)}^{p/q}-{\left(\lambda_{Z}\left(g\right)\right)}^{p/q}\right)}^{q/p}$*. For simplicity, a pair* (*γ_G_*, *λ_G_*) *is referred to as a Fractional orthopair fuzzy number (FOFN), denoted by ϑ* = (*γ_ϑ_*, *λ_ϑ_*).

**Definition** **2** ([40])**.** *Let ϑ*_1_ = (*γ*_1_, *λ*_1_) *and ϑ*_2_ = (*γ*_2_, *λ*_2_) *be two FOFNs and ζ* > 0*, then*

*(i)*  $\vartheta_{1}⊕\vartheta_{2}=\left({\left(\gamma_{1}^{p/q}+\gamma_{2}^{p/q}-\gamma_{1}^{p/q}\gamma_{2}^{p/q}\right)}^{q/p},\lambda_{1}\lambda_{2}\right)$*,*
*(ii)*  $\vartheta_{1}⊗\vartheta_{2}=\left(\gamma_{1}\gamma_{2},{\left(\lambda_{1}^{p/q}+\lambda_{2}^{p/q}-\lambda_{1}^{p/q}\lambda_{2}^{p/q}\right)}^{q/p}\right)$*,*
*(iii)*  $\zeta \vartheta_{1}={\left(1-{\left(1-\gamma_{1}^{p/q}\right)}^{\zeta },\lambda_{1}^{\zeta }\right)}^{q/p}$*,*
*(iv)*  $\vartheta_{1}^{\zeta }=\left(\gamma_{1}^{\zeta },{\left(1-{\left(1-\lambda_{1}^{p/q}\right)}^{\zeta }\right)}^{q/p}\right)$*.*

*For any two positive real numbers ζ*_1_ *and ζ*_2_ *, the operational laws defined in Definition 2 satisfy the following properties:*

*(i)*  *ϑ*_1_ ⊕ *ϑ*_2_ = *ϑ*_2_ ⊕ *ϑ*_1_,
*(ii)*  *ζ*_1_(*ϑ*_1_ ⊕ *ϑ*_2_) = *ζ*_1_*ϑ*_1_ ⊕ *ζ*_1_*ϑ*_2_,
*(iii)*  ${\left(\vartheta_{1}⊗\vartheta_{2}\right)}^{\zeta_{1}}=\vartheta_{1}^{\zeta_{1}}⊗\vartheta_{2}^{\zeta_{1}}$,
*(iv)*  $\vartheta_{1}^{\zeta_{1}+\zeta_{1}}=\vartheta_{1}^{\zeta_{1}}⊗\vartheta_{2}^{\zeta_{1}}$.

**Definition** **3** ([40])**.** *Let ϑ* = (*γ*, *λ*)*, be a FOFNs, then the score function* (*S*) *of ϑ is defined as* $S\left(\vartheta \right)=\frac{1+\gamma^{p/q}-\lambda^{p/q}}{2}$*, where* 0 ≤ *S*(*ϑ*) ≤ 1*. Similarly, the accuracy function* (*A*) *of ϑ is defined as A*(*ϑ*) = *γ^p^*^/*q*^ + *λ^p^*^/*q*^*, where* 0 ≤ (*ϑ*) ≤ 1*. For any two FOFNs ϑ*_1_ = (*γ*_1_, *λ*_1_) *and ϑ*_2_ = (*γ*_1_, *λ*_2_)*, the following properties hold true.*

*(i)*  *If S*(*ϑ*_1_) > *S*(*ϑ*_2_) *then ϑ*_1_ > *ϑ*_2_*,*
*(ii)*  *S*(*ϑ*_1_) < *S*(*ϑ*_2_) *then ϑ*_1_ < *ϑ*_2_*,*
*(iii)*  *S*(*ϑ*_1_) = *S*(*ϑ*_2_) *then*
  - *A*(*ϑ*_1_) > *A*(*ϑ*_1_) *then ϑ*_1_ > *ϑ*_2_*,*
  - *A*(*ϑ*_1_) < *A*(*ϑ*_1_) *then ϑ*_1_ < *ϑ*_2_*,*
  - *A*(*ϑ*_1_) = *A*(*ϑ*_1_) *and S*(*ϑ*_1_) = *S*(*ϑ*_2_) *ϑ*_1_ = *ϑ*_2_.

The operational laws introduced by Zhang et al. [40] provide a valid algebraic framework for FOF information, while the choice of a t-norm and t-conorm allows different aggregation behaviors to be modeled. In fuzzy decision-making, this choice affects how membership and non-membership information from different experts interact and, consequently, may influence the resulting scores and rankings. The proposed operations introduce the Einstein t-norm and t-conorm into the fractional-power structure of FOF numbers, providing a different nonlinear aggregation mechanism from the algebraic operations in [40]. Based on these operations, the FOFEWA and FOFEG operators are developed and their commutativity, associativity, monotonicity, and related algebraic properties are established. The proposed operators therefore provide an additional mathematically grounded aggregation mechanism for FOF information, with potentially different ranking and sensitivity behavior under the same expert assessments.

## 3. Proposed Operational Laws and Aggregation Operators

In this section, we introduce new operational laws for FOFNs based on the classical Einstein (t)-norm and (t)-conorm, which provide the underlying mathematical foundation for the proposed operations. The classical Einstein *t*-norm and *t*-conorm for *α*, *β* ∈ [0,1] are defined by Equations (2) and (3), respectively. These fundamental operations are subsequently extended to the FOFN environment to formulate the proposed operational laws.

- Einstein t-norm

(2) $$\tau_{E}(\alpha ,\beta )=\frac{\alpha \beta }{2-\alpha -\beta +\alpha \beta }$$

- Einstein t-conorm

(3) $$\sigma_{E}(\alpha ,\beta )=\frac{\alpha +\beta }{1+\alpha \beta }$$

They satisfy the standard duality relation *σ_E_*(*α*, *β*) = 1 − *τ_E_*(1 − *α*,1 − *β*).

### 3.1. New Operations for FOFSs Based on Einstein Norms

**Definition** **4.** *Consider ϑ*_1_ = (*γ*_1_, *λ*_1_) *and ϑ*_2_ = (*γ*_2_, *λ*_2_)*, be any two FOFNs and ζ* > 0*, then Einstein operations for the FOFNs are defined as follows:*

*(i)*  $\vartheta_{1}⊕\vartheta_{2}=\left({\left(\frac{\gamma_{1}^{p/q}+\gamma_{2}^{p/q}}{1+\gamma_{1}^{p/q}\gamma_{2}^{p/q}}\right)}^{q/p},\frac{\lambda_{1}\lambda_{2}}{{\left(1+\left(1-\lambda_{1}^{p/q}\right)\left(1-\lambda_{2}^{p/q}\right)\right)}^{q/p}}\right)$*,*
*(ii)*  $\vartheta_{1}⊗\vartheta_{2}=\left(\frac{\gamma_{1}^{p/q}+\gamma_{2}^{p/q}}{{\left(1+\left(1-\gamma_{1}^{p/q}\right)\left(1-\gamma_{2}^{p/q}\right)\right)}^{q/p}},{\left(\frac{\lambda_{1}^{p/q}+\lambda_{2}^{p/q}}{1+\lambda_{1}^{p/q}\lambda_{2}^{p/q}},\right)}^{q/p}\right)$*,*
*(iii)*  $\zeta \vartheta_{1}=\left({\left(\frac{{\left(1+\gamma_{1}^{p/q}\right)}^{\zeta }-{\left(1-\gamma_{1}^{p/q}\right)}^{\zeta }}{{\left(1+\gamma_{1}^{p/q}\right)}^{\zeta }+{\left(1-\gamma_{1}^{p/q}\right)}^{\zeta }}\right)}^{q/p},\frac{{\left(2\right)}^{q/p}{\left(\lambda_{1}\right)}^{\zeta }}{{\left({\left(2-\lambda_{1}^{p/q}\right)}^{\zeta }+{\left(\lambda_{1}^{p/q}\right)}^{\zeta }\right)}^{q/p}}\right)$*,*
*(iv)*  $\vartheta_{1}^{\zeta }=\left(\frac{{\left(2\right)}^{q/p}{\left(\gamma_{1}\right)}^{\zeta }}{{\left({\left(2-\gamma_{1}^{p/q}\right)}^{\zeta }+{\left(\gamma_{1}^{p/q}\right)}^{\zeta }\right)}^{q/p}},{\left(\frac{{\left(1+\lambda_{1}^{p/q}\right)}^{\zeta }-{\left(1-\lambda_{1}^{p/q}\right)}^{\zeta }}{{\left(1+\lambda_{1}^{p/q}\right)}^{\zeta }+{\left(1-\lambda_{1}^{p/q}\right)}^{\zeta }}\right)}^{q/p}\right)$*.*

**Theorem** **1.** *Consider ϑ*_1_ = (*γ*_1_, *λ*_1_) *and ϑ*_2_ = (*γ*_2_, *λ*_2_)*, be any two FOFNs and ζ*_1_*, ζ*_2_ *be any two positive real numbers, then*

*(i)*  *ϑ*_1_ ⊕ *ϑ*_2_ = *ϑ*_2_ ⊕ *ϑ*_1_*,*
*(ii)*  *ϑ*_1_ ⊗ *ϑ*_2_ = *ϑ*_2_ ⊗ *ϑ*_1_*,*
*(iii)*  $\zeta_{1}\left(\vartheta_{1}⊕\vartheta_{2}\right)=\zeta_{1}\vartheta_{1}⊕{\zeta_{1}\vartheta }_{2}$*,*
*(iv)*  $\vartheta_{1}^{\zeta_{1}+\zeta_{2}}=\vartheta_{1}^{\zeta_{1}}⊗\vartheta_{1}^{\zeta_{2}}$*,*
*(v)*  $\zeta_{1}\left(\vartheta_{1}⊗\vartheta_{2}\right)=\zeta_{1}\vartheta_{1}⊗{\zeta_{1}\vartheta }_{2}$*,*
*(vi)*  ${\left(\vartheta_{1}⊗\vartheta_{2}\right)}^{\zeta_{1}}=\vartheta_{1}^{\zeta_{1}}⊗\vartheta_{2}^{\zeta_{1}}$*,*
*(vii)*  $\vartheta_{1}\left(\zeta_{1}+\zeta_{2}\right)=\zeta_{1}\vartheta_{1}⊗{\zeta_{1}\vartheta }_{1}$*.*

**Proof.**  (i) According to Definition 4, we obtain

$$\vartheta_{1}⊕\vartheta_{2}=\left({\left(\frac{\gamma_{1}^{p/q}+\gamma_{2}^{p/q}}{1+\gamma_{1}^{p/q}\gamma_{2}^{p/q}},\right)}^{q/p},\frac{\lambda_{1}\lambda_{2}}{{\left(1+\left(1-\lambda_{1}^{p/q}\right)\left(1-\lambda_{2}^{p/q}\right)\right)}^{q/p}}\right)$$

$$=\left({\left(\frac{\gamma_{2}^{p/q}+\gamma_{1}^{p/q}}{1+\gamma_{2}^{p/q}\gamma_{1}^{p/q}},\right)}^{q/p},\frac{\lambda_{2}\lambda_{1}}{{\left(1+\left(1-\lambda_{2}^{p/q}\right)\left(1-\lambda_{1}^{p/q}\right)\right)}^{q/p}}\right)=\vartheta_{2}⊕\vartheta_{1}$$

The second part of Theorem 1 follows from an analogous argument.

(ii) Let $M=\left(1+\gamma_{1}^{p/q}\right)\left(1+\gamma_{2}^{p/q}\right)$, $N=\left(1-\gamma_{1}^{p/q}\right)\left(1-\gamma_{2}^{p/q}\right)$, $T=\lambda_{1}^{p/q}\lambda_{2}^{p/q}$ and $P=\left(2-\lambda_{1}^{p/q}\right)\left(2-\lambda_{2}^{p/q}\right)$, then

$$\vartheta_{1}⊕\vartheta_{2}=\left({\left(\frac{M-N}{M+N}\right)}^{q/p},\frac{{\left(2T\right)}^{q/p}}{{\left(2T+P\right)}^{q/p}}\right)$$

and

$$\zeta \left(\vartheta_{1}⊕\vartheta_{2}\right)=\zeta \left({\left(\frac{M-N}{M+N}\right)}^{q/p},\frac{{\left(2T\right)}^{q/p}}{{\left(2T+P\right)}^{q/p}}\right)$$

$$=\left(\frac{{\left({\left(1+\frac{M-N}{M+N}\right)}^{\zeta }\right)}^{q/p}-{\left({\left(1-\frac{M-N}{M+N}\right)}^{\zeta }\right)}^{q/p}}{{\left({\left(1+\frac{M-N}{M+N}\right)}^{\zeta }\right)}^{q/p}+{\left({\left(1-\frac{M-N}{M+N}\right)}^{\zeta }\right)}^{q/p}},\frac{{\left(2\right)}^{q/p}\frac{{\left(2T\right)}^{q/p}}{{\left(2T+P\right)}^{q/p}}}{{\left({\left(2\right)}^{q/p}-\frac{{\left(2T\right)}^{q/p}}{{\left(2T+P\right)}^{q/p}}\right)}^{\zeta }+{\left(\frac{{\left(2T\right)}^{q/p}}{{\left(2T+P\right)}^{q/p}}\right)}^{\zeta }}\right)$$

$$=\left({\left(\frac{M^{\zeta }-N^{\zeta }}{M^{\zeta }+N^{\zeta }}\right)}^{q/p},\frac{{\left(2T^{\zeta }\right)}^{q/p}}{{\left(T^{\zeta }+P^{\zeta }\right)}^{q/p}}\right)$$

$$=\left({\left(\frac{{\left(1+\gamma_{1}^{p/q}\right)}^{\zeta }-{\left(1-\gamma_{1}^{p/q}\right)}^{\zeta }}{{\left(1+\gamma_{1}^{p/q}\right)}^{\zeta }+{\left(1-\gamma_{1}^{p/q}\right)}^{\zeta }}\right)}^{q/p},\frac{{\left(2\right)}^{q/p}{\left(\lambda_{1}\right)}^{\zeta }}{{\left({\left(2-\lambda_{1}^{p/q}\right)}^{\zeta }+{\left(\lambda_{1}^{p/q}\right)}^{\zeta }\right)}^{q/p}}\right).$$

Consider $M_{1}={\left(1+\gamma_{1}^{p/q}\right)}^{\zeta }$, ${N_{1}=\left(1-\gamma_{1}^{p/q}\right)}^{\zeta }$, $T_{1}={\left(\lambda_{1}^{p/q}\right)}^{\zeta }$, $P_{1}={\left(2-\lambda_{1}^{p/q}\right)}^{\zeta }$, $M_{2}={\left(2+\gamma_{2}^{p/q}\right)}^{\zeta }$, $N_{2}={\left(2-\gamma_{2}^{p/q}\right)}^{\zeta }$, $T_{2}={\left(\lambda_{2}^{p/q}\right)}^{\zeta }$ and $P_{2}={\left(2-\lambda_{2}^{p/q}\right)}^{\zeta }$, then

$$\begin{array}{c} \zeta \vartheta_{1}=\left({\left(\frac{{\left(1+\gamma_{1}^{p/q}\right)}^{\zeta }-{\left(1-\gamma_{1}^{p/q}\right)}^{\zeta }}{{\left(1+\gamma_{1}^{p/q}\right)}^{\zeta }+{\left(1-\gamma_{1}^{p/q}\right)}^{\zeta }}\right)}^{q/p},\frac{{\left(2\right)}^{q/p}{\left(\lambda_{1}\right)}^{\zeta }}{{\left({\left(2-\lambda_{1}^{p/q}\right)}^{\zeta }+{\left(\lambda_{1}^{p/q}\right)}^{\zeta }\right)}^{q/p}}\right)\\ =\left({\left(\frac{M_{1}-N_{1}}{M_{1}+N_{1}}\right)}^{q/p},\frac{{\left(2T_{1}\right)}^{q/p}}{{\left(2T_{1}+P_{1}\right)}^{q/p}}\right) \end{array}$$

$$\begin{array}{c} \zeta \vartheta_{2}=\left({\left(\frac{{\left(1+\gamma_{2}^{p/q}\right)}^{\zeta }-{\left(1-\gamma_{2}^{p/q}\right)}^{\zeta }}{{\left(1+\gamma_{2}^{p/q}\right)}^{\zeta }+{\left(1-\gamma_{2}^{p/q}\right)}^{\zeta }}\right)}^{q/p},\frac{{\left(2\right)}^{q/p}{\left(\lambda_{2}\right)}^{\zeta }}{{\left({\left(2-\lambda_{2}^{p/q}\right)}^{\zeta }+{\left(\lambda_{2}^{p/q}\right)}^{\zeta }\right)}^{q/p}}\right)\\ =\left({\left(\frac{M_{2}-N_{2}}{M_{2}+N_{2}}\right)}^{q/p},\frac{{\left(2T_{2}\right)}^{q/p}}{{\left(2T_{2}+P_{2}\right)}^{q/p}}\right). \end{array}$$

Based on Definition 4, we obtain

$$\zeta \vartheta_{1}⊕{\zeta \vartheta }_{2}=\left({\left(\frac{M_{1}-N_{1}}{M_{1}+N_{1}}\right)}^{q/p},\frac{{\left(2T_{1}\right)}^{q/p}}{{\left(2T_{1}+P_{1}\right)}^{q/p}}\right)⊕\left({\left(\frac{M_{2}-N_{2}}{M_{2}+N_{2}}\right)}^{q/p},\frac{{\left(2T_{2}\right)}^{q/p}}{{\left(2T_{2}+P_{2}\right)}^{q/p}}\right)$$

$$=\left({\left(\frac{\frac{M_{1}-N_{1}}{M_{1}+N_{1}}+\frac{M_{2}-N_{2}}{M_{2}+N_{2}}}{1+\left(\frac{M_{1}-N_{1}}{M_{1}+N_{1}}\right)\left(\frac{M_{2}-N_{2}}{M_{2}+N_{2}}\right)}\right)}^{q/p},\frac{2{\left(T_{1}T_{2}\right)}^{q/p}}{{1+\left(\left(1-\frac{{\left(2T_{1}\right)}^{q/p}}{{\left(T_{1}+P_{1}\right)}^{q/p}}\right)\left(1-\frac{{\left(2T_{2}\right)}^{q/p}}{{\left(T_{2}+P_{2}\right)}^{q/p}}\right)\right)}^{q/p}}\right)$$

$$=\left({\left(\frac{M_{1}M_{2}-N_{1}N_{2}}{M_{1}M_{2}+N_{1}N_{2}}\right)}^{q/p},\frac{2{\left(T_{1}T_{2}\right)}^{q/p}}{{\left(T_{1}T_{2}+{P_{1}P}_{2}\right)}^{q/p}}\right)$$

$$=\left(\begin{array}{c} {\left(\frac{{\left(1+\gamma_{1}^{p/q}\right)}^{\zeta }{\left(1+\gamma_{2}^{p/q}\right)}^{\zeta }-{\left(1-\gamma_{1}^{p/q}\right)}^{\zeta }{\left(1-\gamma_{2}^{p/q}\right)}^{\zeta }}{{\left(1+\gamma_{1}^{p/q}\right)}^{\zeta }{\left(1+\gamma_{2}^{p/q}\right)}^{\zeta }+{\left(1-\gamma_{1}^{p/q}\right)}^{\zeta }{\left(1-\gamma_{2}^{p/q}\right)}^{\zeta }}\right)}^{q/p},\\ \frac{{\left(2\right)}^{q/p}{\left(\lambda_{1}\right)}^{\zeta }{\left(\lambda_{2}\right)}^{\zeta }}{{\left({\left(2-\lambda_{1}^{p/q}\right)}^{\zeta }{\left(2-\lambda_{2}^{p/q}\right)}^{\zeta }+{\left(\lambda_{1}^{p/q}\right)}^{\zeta }{\left(\lambda_{2}^{p/q}\right)}^{\zeta }\right)}^{q/p}} \end{array}\right).$$

Hence *ζ*(*ϑ*_1_ ⊕ *ϑ*_2_) = *ζϑ*_1_ ⊕ *ζϑ*_2_.

(iii) According to Definition 4, we have

$$\zeta_{1}\vartheta_{1}=\left({\left(\frac{{\left(1+\gamma_{1}^{p/q}\right)}^{\zeta_{1}}-{\left(1-\gamma_{1}^{p/q}\right)}^{\zeta_{1}}}{{\left(1+\gamma_{1}^{p/q}\right)}^{\zeta_{1}}+{\left(1-\gamma_{1}^{p/q}\right)}^{\zeta_{1}}}\right)}^{q/p},\frac{{\left(2\right)}^{q/p}{\left(\lambda_{1}\right)}^{\zeta_{1}}}{{\left({\left(2-\lambda_{1}^{p/q}\right)}^{\zeta_{1}}+{\left(\lambda_{1}^{p/q}\right)}^{\zeta_{1}}\right)}^{q/p}}\right),$$

$$\zeta_{2}\vartheta_{1}=\left({\left(\frac{{\left(1+\gamma_{1}^{p/q}\right)}^{\zeta_{2}}-{\left(1-\gamma_{1}^{p/q}\right)}^{\zeta_{2}}}{{\left(1+\gamma_{1}^{p/q}\right)}^{\zeta_{2}}+{\left(1-\gamma_{1}^{p/q}\right)}^{\zeta_{2}}}\right)}^{q/p},\frac{{\left(2\right)}^{q/p}{\left(\lambda_{1}\right)}^{\zeta_{2}}}{{\left({\left(2-\lambda_{1}^{p/q}\right)}^{\zeta_{2}}+{\left(\lambda_{1}^{p/q}\right)}^{\zeta_{2}}\right)}^{q/p}}\right).$$

Then,

$$\zeta_{1}\vartheta_{1}⊕{\zeta_{2}\vartheta }_{1}=\left({\left(\frac{{\left(1+\gamma_{1}^{p/q}\right)}^{\zeta_{1}}-{\left(1-\gamma_{1}^{p/q}\right)}^{\zeta_{1}}}{{\left(1+\gamma_{1}^{p/q}\right)}^{\zeta_{1}}+{\left(1-\gamma_{1}^{p/q}\right)}^{\zeta_{1}}}\right)}^{q/p},\frac{{\left(2\right)}^{q/p}{\left(\lambda_{1}\right)}^{\zeta_{1}}}{{\left({\left(2-\lambda_{1}^{p/q}\right)}^{\zeta_{1}}+{\left(\lambda_{1}^{p/q}\right)}^{\zeta_{1}}\right)}^{q/p}}\right)$$

$$⊕\left({\left(\frac{{\left(1+\gamma_{1}^{p/q}\right)}^{\zeta_{2}}-{\left(1-\gamma_{1}^{p/q}\right)}^{\zeta_{2}}}{{\left(1+\gamma_{1}^{p/q}\right)}^{\zeta_{2}}+{\left(1-\gamma_{1}^{p/q}\right)}^{\zeta_{2}}}\right)}^{q/p},\frac{{\left(2\right)}^{q/p}{\left(\lambda_{1}\right)}^{\zeta_{2}}}{{\left({\left(2-\lambda_{1}^{p/q}\right)}^{\zeta_{2}}+{\left(\lambda_{1}^{p/q}\right)}^{\zeta_{2}}\right)}^{q/p}}\right)$$

$$=\left(\begin{array}{c} {\left(\frac{{\left(1+\gamma_{1}^{p/q}\right)}^{\zeta_{1}+\zeta_{2}}-{\left(1-\gamma_{1}^{p/q}\right)}^{\zeta_{1}+\zeta_{2}}}{{\left(1+\gamma_{1}^{p/q}\right)}^{\zeta_{1}+\zeta_{2}}+{\left(1-\gamma_{1}^{p/q}\right)}^{\zeta_{1}+\zeta_{2}}}\right)}^{q/p}\\ ,\frac{{\left(2\right)}^{q/p}{\left(\lambda_{1}\right)}^{\zeta_{1}+\zeta_{2}}}{{\left({\left(2-\lambda_{1}^{p/q}\right)}^{\zeta_{1}+\zeta_{2}}+{\left(\lambda_{1}^{p/q}\right)}^{\zeta_{1}+\zeta_{2}}\right)}^{q/p}} \end{array}\right)=\left(\zeta_{1}+\zeta_{2}\right)\vartheta_{1}.$$

The remaining part follows from an analogous argument. □

### 3.2. Fractional Orthopair Fuzzy Einstein Aggregation Operators

**Definition** **5.** *Let ϑ_i_* = (*γ_i_*, *λ_i_*)*,* (*i* = 1, 2, …, *k*) *denote a collection of FOFNs associated with weights ϖ_i_, where ϖ_i_* ∈ [0,1] *and* $\sum_{i=1}^{k}\varpi_{i}=1$*. The fractional orthopair fuzzy Einstein weighted averaging operator, abbreviated as FOFEWA*(*ϑ*_1_, *ϑ*_2_, …, *ϑ_k_*)*, is a function mapping Δ^k^ into Δ, and is given by:*

(4) $$\mathrm{FOFEWA}\left(\vartheta_{1},\vartheta_{2},\ldots ,\vartheta_{k}\right)={⊕}_{i=1}^{k}\varpi_{i}\vartheta_{i}$$

**Theorem** **2.** *The result obtained using the FOFEWA operator remains a FOFN.*

(5) $$\mathrm{FOFEWA}\left(\vartheta_{1},\vartheta_{2},\ldots ,\vartheta_{k}\right)=\left(\begin{array}{c} {\left(\frac{{\prod_{i=1}^{k}\left(1+\gamma_{i}^{p/q}\right)}^{\varpi_{i}}-{\prod_{i=1}^{k}\left(1-\gamma_{i}^{p/q}\right)}^{\varpi_{i}}}{{\prod_{i=1}^{k}\left(1+\gamma_{i}^{p/q}\right)}^{\varpi_{i}}+{\prod_{i=1}^{k}\left(1-\gamma_{i}^{p/q}\right)}^{\varpi_{i}}}\right)}^{q/p}\\ ,\frac{{\left(2\right)}^{q/p}{\prod_{i=1}^{k}\left(\lambda_{i}^{p/q}\right)}^{\varpi_{i}}}{{\prod_{i=1}^{k}\left(2-\lambda_{i}^{p/q}\right)}^{\varpi_{i}}+\prod_{i=1}^{k}{\left(\lambda_{i}^{p/q}\right)}^{\varpi_{i}}} \end{array}\right)$$

**Proof.**  The proof of Theorem 2 proceeds by mathematical induction.

**Step 1.** If *k* = 2, then FOFEWA$\left(\vartheta_{1},\vartheta_{2}\right)={⊕}_{i=1}^{2}\varpi_{i}\vartheta_{i}=\varpi_{1}\vartheta_{1}⊕\varpi_{2}\vartheta_{2}$. According to Definition 4, we obtain

$$\varpi_{1}\vartheta_{1}=\left({\left(\frac{{\left(1+\gamma_{1}^{p/q}\right)}^{\varpi_{1}}-{\left(1-\gamma_{1}^{p/q}\right)}^{\varpi_{1}}}{{\left(1+\gamma_{1}^{p/q}\right)}^{\varpi_{1}}+{\left(1-\gamma_{1}^{p/q}\right)}^{\varpi_{1}}}\right)}^{q/p},\frac{{\left(2\right)}^{q/p}{\left(\lambda_{1}\right)}^{\varpi_{1}}}{{\left({\left(2-\lambda_{1}^{p/q}\right)}^{\varpi_{1}}+{\left(\lambda_{1}^{p/q}\right)}^{\varpi_{1}}\right)}^{q/p}}\right),$$

$$\varpi_{2}\vartheta_{2}=\left({\left(\frac{{\left(1+\gamma_{2}^{p/q}\right)}^{\varpi_{2}}-{\left(1-\gamma_{2}^{p/q}\right)}^{\varpi_{2}}}{{\left(1+\gamma_{2}^{p/q}\right)}^{\varpi_{2}}+{\left(1-\gamma_{2}^{p/q}\right)}^{\varpi_{2}}}\right)}^{q/p},\frac{{\left(2\right)}^{q/p}{\left(\lambda_{2}\right)}^{\varpi_{2}}}{{\left({\left(2-\lambda_{2}^{p/q}\right)}^{\varpi_{2}}+{\left(\lambda_{2}^{p/q}\right)}^{\varpi_{2}}\right)}^{q/p}}\right).$$

Then,

$$\varpi_{1}\vartheta_{1}⊕\varpi_{2}\vartheta_{2}=\left({\left(\frac{{\left(1+\gamma_{1}^{p/q}\right)}^{\varpi_{1}}-{\left(1-\gamma_{1}^{p/q}\right)}^{\varpi_{1}}}{{\left(1+\gamma_{1}^{p/q}\right)}^{\varpi_{1}}+{\left(1-\gamma_{1}^{p/q}\right)}^{\varpi_{1}}}\right)}^{q/p},\frac{{\left(2\right)}^{q/p}{\left(\lambda_{1}\right)}^{\varpi_{1}}}{{\left({\left(2-\lambda_{1}^{p/q}\right)}^{\varpi_{1}}+{\left(\lambda_{1}^{p/q}\right)}^{\varpi_{1}}\right)}^{q/p}}\right)$$

$$⊕\left({\left(\frac{{\left(1+\gamma_{2}^{p/q}\right)}^{\varpi_{2}}-{\left(1-\gamma_{2}^{p/q}\right)}^{\varpi_{2}}}{{\left(1+\gamma_{2}^{p/q}\right)}^{\varpi_{2}}+{\left(1-\gamma_{2}^{p/q}\right)}^{\varpi_{2}}}\right)}^{q/p},\frac{{\left(2\right)}^{q/p}{\left(\lambda_{2}\right)}^{\varpi_{2}}}{{\left({\left(2-\lambda_{2}^{p/q}\right)}^{\varpi_{2}}+{\left(\lambda_{2}^{p/q}\right)}^{\varpi_{2}}\right)}^{q/p}}\right)$$

$$=\left(\begin{array}{c} {\left(\frac{{\prod_{i=1}^{2}\left(1+\gamma_{i}^{p/q}\right)}^{\varpi_{i}}-{\prod_{i=1}^{2}\left(1-\gamma_{i}^{p/q}\right)}^{\varpi_{i}}}{{\prod_{i=1}^{2}\left(1+\gamma_{i}^{p/q}\right)}^{\varpi_{i}}+{\prod_{i=1}^{k}\left(1-\gamma_{i}^{p/q}\right)}^{\varpi_{i}}}\right)}^{q/p}\\ ,\frac{{\left(2\right)}^{q/p}{\prod_{i=1}^{2}\left(\lambda_{i}^{p/q}\right)}^{\varpi_{i}}}{{\prod_{i=1}^{2}\left(2-\lambda_{i}^{p/q}\right)}^{\varpi_{i}}+\prod_{i=1}^{2}{\left(\lambda_{i}^{p/q}\right)}^{\varpi_{i}}} \end{array}\right).$$

where $\sum_{i=1}^{2}\varpi_{i}=1$, therefore the results are true for *k* = 2.

**Step 2.** Suppose the statement is valid for *k* = *m*. It follows that

$$\mathrm{FOFWA}\left(\vartheta_{1},\vartheta_{2},\ldots ,\vartheta_{m}\right)={⊕}_{i=1}^{m}\varpi_{i}\vartheta_{i}$$

(6) $$=\left(\begin{array}{c} {\left(\frac{{\prod_{i=1}^{m}\left(1+\gamma_{i}^{p/q}\right)}^{\varpi_{i}}-{\prod_{i=1}^{m}\left(1-\gamma_{i}^{p/q}\right)}^{\varpi_{i}}}{{\prod_{i=1}^{m}\left(1+\gamma_{i}^{p/q}\right)}^{\varpi_{i}}+{\prod_{i=1}^{m}\left(1-\gamma_{i}^{p/q}\right)}^{\varpi_{i}}}\right)}^{q/p}\\ ,\frac{{\left(2\right)}^{q/p}{\prod_{i=1}^{m}\left(\lambda_{i}^{p/q}\right)}^{\varpi_{i}}}{{\prod_{i=1}^{m}\left(2-\lambda_{i}^{p/q}\right)}^{\varpi_{i}}+\prod_{i=1}^{m}{\left(\lambda_{i}^{p/q}\right)}^{\varpi_{i}}} \end{array}\right)$$

**Step 3.** We have to prove that Equation (5), is true for *k* = *m* + 1, i.e.,

$$\mathrm{FOFEWA}\left(\vartheta_{1},\vartheta_{2},\ldots ,\vartheta_{m+1}\right)={⊕}_{i=1}^{m+1}\varpi_{i}\vartheta_{i}={⊕}_{i=1}^{m}\varpi_{i}\vartheta_{i}⊕\varpi_{m+1}\varpi_{m+1}.$$

By applying Equation (4), we have ${⊕}_{i=1}^{m+1}\varpi_{i}\vartheta_{i}={⊕}_{i=1}^{m}\varpi_{i}\vartheta_{i}⊕\varpi_{m+1}\vartheta_{m+1}$

$$=\left({\left(\frac{{\prod_{i=1}^{m}\left(1+\gamma_{i}^{p/q}\right)}^{\varpi_{i}}-{\prod_{i=1}^{m}\left(1-\gamma_{i}^{p/q}\right)}^{\varpi_{i}}}{{\prod_{i=1}^{m}\left(1+\gamma_{i}^{p/q}\right)}^{\varpi_{i}}+{\prod_{i=1}^{m}\left(1-\gamma_{i}^{p/q}\right)}^{\varpi_{i}}}\right)}^{q/p},\frac{{\left(2\right)}^{q/p}{\prod_{i=1}^{m}\left(\lambda_{i}^{p/q}\right)}^{\varpi_{i}}}{{\prod_{i=1}^{m}\left(2-\lambda_{i}^{p/q}\right)}^{\varpi_{i}}+\prod_{i=1}^{m}\left(\lambda_{i}^{\varpi_{i}}\right)}\right)$$

$$⊕\left({\left(\frac{{\left(1+\gamma_{m+1}^{p/q}\right)}^{\varpi_{m+1}}-{\left(1-\gamma_{m+1}^{p/q}\right)}^{\varpi_{m+1}}}{{\left(1+\gamma_{m+1}^{p/q}\right)}^{\varpi_{m+1}}+{\left(1-\gamma_{m+1}^{p/q}\right)}^{\varpi_{m+1}}}\right)}^{q/p},\frac{{\left(2\right)}^{q/p}{\left(\lambda_{m+1}\right)}^{\varpi_{m+1}}}{{\left({\left(2-\lambda_{m+1}^{p/q}\right)}^{\varpi_{m+1}}+{\left(\lambda_{m+1}^{p/q}\right)}^{\varpi_{m+1}}\right)}^{q/p}}\right)$$

$$=\left({\left(\frac{{\prod_{i=1}^{m+1}\left(1+\gamma_{i}^{p/q}\right)}^{\varpi_{i}}-{\prod_{i=1}^{m+1}\left(1-\gamma_{i}^{p/q}\right)}^{\varpi_{i}}}{{\prod_{i=1}^{m+1}\left(1+\gamma_{i}^{p/q}\right)}^{\varpi_{i}}+{\prod_{i=1}^{m+1}\left(1-\gamma_{i}^{p/q}\right)}^{\varpi_{i}}}\right)}^{q/p},\frac{{\left(2\right)}^{q/p}{\prod_{i=1}^{m+1}\left(\lambda_{i}^{p/q}\right)}^{\varpi_{i}}}{{\prod_{i=1}^{m+1}\left(2-\lambda_{i}^{p/q}\right)}^{W_{i}}+\prod_{i=1}^{m+1}{\left(\lambda_{i}^{p/q}\right)}^{\varpi_{i}}}\right).$$

where $\sum_{i=1}^{m+1}\varpi_{i}=1$, therefore, Equation (5) is true for *k* = *m* + 1. □

**Theorem** **3.** *Let ϑ_i_* = (*γ_i_*, *λ_i_*)*,* (*i* = 1, 2, …, *k*) *are identical, i.e., ϑ_i_* = *ϑ for all i. Then FOFEWA*(*ϑ*_1_, *ϑ*_2_, …, *ϑ_k_*) = *ϑ.*

**Proof.**  Given that *ϑ_i_* = *ϑ* for all *i*, this implies that

$$\mathrm{FOFEWA}\left(E_{1},E_{2},\ldots ,E_{k}\right)=\left({\left(\frac{{\prod_{i=1}^{k}\left(1+\gamma_{i}^{p/q}\right)}^{\varpi_{i}}-{\prod_{i=1}^{k}\left(1-\gamma_{i}^{p/q}\right)}^{\varpi_{i}}}{{\prod_{i=1}^{k}\left(1+\gamma_{i}^{p/q}\right)}^{\varpi_{i}}+{\prod_{i=1}^{k}\left(1-\gamma_{i}^{p/q}\right)}^{\varpi_{i}}}\right)}^{q/p},\frac{{\left(2\right)}^{q/p}{\prod_{i=1}^{k}\left(\lambda_{i}^{p/q}\right)}^{\varpi_{i}}}{{\prod_{i=1}^{k}\left(2-\lambda_{i}^{p/q}\right)}^{W_{i}}+\prod_{i=1}^{k}\left(\lambda_{i}^{\varpi_{i}}\right)}\right)$$

$$=\left({\left(\frac{{\left(1+\gamma_{i}^{p/q}\right)}^{\sum_{i=1}^{k}\varpi_{i}}-{\left(1-\gamma_{i}^{p/q}\right)}^{\sum_{i=1}^{k}\varpi_{i}}}{{\left(1+\gamma_{i}^{p/q}\right)}^{\sum_{i=1}^{k}\varpi_{i}}+{\left(1-\gamma_{i}^{p/q}\right)}^{\sum_{i=1}^{k}\varpi_{i}}}\right)}^{q/p},\frac{{\left(2\right)}^{q/p}{\left(\lambda_{i}^{p/q}\right)}^{\sum_{i=1}^{k}\varpi_{i}}}{{\left(2-\lambda_{i}^{p/q}\right)}^{\sum_{i=1}^{k}\varpi_{i}}+\left(\lambda_{i}^{\sum_{i=1}^{k}\varpi_{i}}\right)}\right)$$

$$=\left({\left(\frac{\left(1+\gamma_{i}^{p/q}\right)-\left(1-\gamma_{i}^{p/q}\right)}{\left(1+\gamma_{i}^{p/q}\right)+\left(1-\gamma_{i}^{p/q}\right)}\right)}^{q/p},\frac{{\left(2\right)}^{q/p}\left(\lambda_{i}^{p/q}\right)}{\left(2-\lambda_{i}^{p/q}\right)+\lambda_{i}}\right)=\left(\gamma ,\lambda \right)=\vartheta .$$

If *ϑ*^−^ = min(*ϑ*_1_, *ϑ*_2_, …, *ϑ_k_*) and *ϑ*^+^ = max(*ϑ*_1_, *ϑ*_2_, …, *ϑ_k_*). Then, the aggregated result obtained by FOFEWA operator satisfies the condition *ϑ*^−^ ≤ *FOFEWA*(*ϑ*_1_, *ϑ*_2_, …, *ϑ_k_*) ≤ *ϑ*^+^. □

**Theorem** **4.** *Let ϑ_i_* = (*γ_i_*, *λ_i_*)*,* (*i* = 1, 2, …, *k*) *and* $\vartheta_{i}^{\prime }=\left(\gamma_{i}^{\prime },\lambda_{i}^{\prime }\right)$*,* (*i* = 1, 2, …, *k*) *be any two collections of FOFNs. If* $\gamma_{i}\leq \gamma_{i}^{\prime }$ *and* $\lambda_{i}\leq \lambda_{i}^{\prime }$*, for all i. Then,* $\mathrm{FOFEWA}\left(\vartheta_{1},\vartheta_{2},\ldots ,\vartheta_{k}\right)\leq \mathrm{FOFEWA}\left(\vartheta_{1}^{\prime },\vartheta_{2}^{\prime },\ldots ,\vartheta_{k}^{\prime }\right)$*.*

**Proof.**  Straightforward. □

**Definition** **6.** *Let E_i_* = (*γ_i_*, *λ_i_*)*,* (*i* = 1, 2, …, *k*) *be a family of FOFNs with corresponding weights ϖ_i_ such that* $\sum_{i=1}^{k}\varpi_{i}=1$ *and ϖ_i_* ∈ [0,1]*. Then the FOFEG*(*ϑ*_1_, *ϑ*_2_, …, *ϑ_k_*) *is mapping from* ∆*^k^* → ∆ *is defined as:*

(7) $$\mathrm{FOFEG}\left(\vartheta_{1},\vartheta_{2},\ldots ,\vartheta_{k}\right)={⊗}_{i=1}^{k}\vartheta_{i}^{\varpi_{i}}$$

**Theorem** **5.** *The result of aggregation under the FOFEG operator remains within the class of FOFNs and is defined as:*

(8) $$\mathrm{FOFEG}\left(\vartheta_{1},\vartheta_{2},\ldots ,\vartheta_{k}\right)\left(\begin{array}{c} \frac{{\left(2\right)}^{q/p}{\prod_{i=1}^{k}\left(\gamma_{i}^{p/q}\right)}^{\varpi_{i}}}{{\prod_{i=1}^{k}\left(2-\gamma_{i}^{p/q}\right)}^{\varpi_{i}}+\prod_{i=1}^{k}\left(\lambda_{i}^{\varpi_{i}}\right)},\\ {\left(\frac{{\prod_{i=1}^{k}\left(1+\lambda_{i}^{p/q}\right)}^{\varpi_{i}}-{\prod_{i=1}^{k}\left(1-\lambda_{i}^{p/q}\right)}^{\varpi_{i}}}{{\prod_{i=1}^{k}\left(1+\lambda_{i}^{p/q}\right)}^{\varpi_{i}}+{\prod_{i=1}^{k}\left(1-\lambda_{i}^{p/q}\right)}^{\varpi_{i}}}\right)}^{q/p} \end{array}\right)$$

**Proof.**  The proof is like that of Theorem 2 and is omitted for brevity. □

The FOFEG operator satisfies the following properties for any set of FOFNs

(i) When all FOFNs *ϑ_i_* = (*γ_i_*, *λ_i_*), *i* = 1, 2, …, *k*, are equal (i.e., *ϑ_i_* = *ϑ* for every *i*), the FOFEG operator returns the same FOFN, namely FOFEG(*ϑ*_1_, *ϑ*_2_, …, *ϑ_k_*) = *ϑ*.
(ii) Let *ϑ_i_* = (*γ_i_*, *λ_i_*), (*i* = 1, 2, …, *k*), be the collection of FOFNs and if *ϑ*^−^ = min(*ϑ*_1_, *ϑ*_2_, …, *ϑ_k_*) and if *ϑ* ^+^ = max(*ϑ*_1_, *ϑ*_2_, …, *ϑ_k_*) then *ϑ*^−^ ≤ *FOFEG*(*ϑ*_1_, *ϑ*_2_, …, *ϑ_k_*) ≤ *ϑ*^+^.
(iii) For any positive real number *ζ*, FOFEG(*ζϑ*_1_, *ζϑ*_2_, …, *ζϑ_k_*) = *ζ*FOFEG(*ϑ*_1_, *ϑ*_2_, …, *ϑ_k_*).
(iv) Consider *ϑ_i_* = (*γ_i_*, *λ_i_*), (*i* = 1, 2, …, *k*) and $\vartheta_{i}^{\prime }=\left(\gamma_{i}^{\prime },\lambda_{i}^{\prime }\right)$, (*i* = 1, 2, …, *k*) be two collections of FOFNs. If $\gamma_{i}\leq \gamma_{i}^{\prime }$ and $\lambda_{i}\geq \lambda_{i}^{\prime }$, then $\mathrm{FOFEG}\left(\vartheta_{1},\vartheta_{2},\ldots ,\vartheta_{k}\right)\leq \mathrm{FOFEWG}\left(\vartheta_{1}^{\prime },\vartheta_{2}^{\prime },\ldots ,\vartheta_{k}^{\prime }\right)$.

**Proof.**  The proof is straightforward. □

**Definition** **7.** *Let ϑ_i_* = (*γ_i_*, *λ_i_*) *(i* = 1,2,3*) be any three FOFNs. Suppose that p* ≥ *q* > 0*, and define r* = *p*/*q*, *s* = *q*/*p*. *Then the distance measure for FOFNs is defined as follows:* $D\left(\vartheta_{1},\vartheta_{2}\right)=\frac{1}{2}\sum_{j=1}^{n}\left[{|\gamma_{1}^{\;r}-{\left(\gamma_{2}^{r}\right)}^{r}|}^{s}+{|\lambda_{1}^{\;r}-{\left(\lambda_{2}^{r}\right)}^{r}|}^{s}\right]$*.*

The proposed distance measure satisfies the following properties:

(i) 𝒟(*ϑ*_1_, *ϑ*_2_) ≥ 0;
(ii) 𝒟(*ϑ*_1_, *ϑ*_2_) = 0 if and only if *ϑ*_1_ = *ϑ*_2_;
(iii) 𝒟(*ϑ*_1_, *ϑ*_2_) = 𝒟(*ϑ*_2_, *ϑ*_1_);
(iv) for any three FOFNs *ϑ*_1_, *ϑ*_2_, *ϑ*_3_, 𝒟(*ϑ*_1_, *ϑ*_3_) ≤ 𝒟(*ϑ*_1_, *ϑ*_2_) + 𝒟(*ϑ*_2_, *ϑ*_3_).

**Proof.**  (i) Since *p* ≥ *q* > 0, we have *r* ≥ 1, 0 < s ≤ 1. For the non-negativity property, since ${|\gamma_{1}^{\;r}-\gamma_{2}^{\;r}|}^{s}\geq 0$ and ${|\lambda_{1}^{\;r}-\lambda_{2}^{\;r}|}^{s}\geq 0,$ it follows directly that 𝒟(*ϑ*_1_, *ϑ*_2_) ≥ 0.

(ii) Suppose that *D*(*ϑ*_1_, *ϑ*_2_) = 0. Because every term in the summation is non-negative, we must have ${|\gamma_{1}^{\;r}-\gamma_{2}^{\;r}|}^{s}=0$ and ${|\lambda_{1}^{\;r}-\lambda_{2}^{\;r}|}^{s}=0$, Hence, $\gamma_{1}^{\;r}=\gamma_{2}^{\;r},\lambda_{1}^{\;r}=\lambda_{2}^{\;r}.$ Since *r* > 0, the function *x* ↦ *x^r^* is strictly increasing on [0, 1]. Therefore, *γ*_1_ = *γ*_2_, *λ*_1_ = *λ*_2_ and consequently *ϑ*_1_ = *ϑ*_2_. Conversely, if *ϑ*_1_ = *ϑ*_2_, then all terms in the definition of 𝒟(*ϑ*_1_, *ϑ*_2_) are zero. Thus, 𝒟(*ϑ*_1_, *ϑ*_2_) = 0  ⟺  *ϑ*_1_ = *ϑ*_2_.

(iii) For symmetry, we have $D\left(\vartheta_{1},\vartheta_{2}\right)=\frac{1}{2}\sum_{j=1}^{n}\left[{|\gamma_{1}^{\;r}-\gamma_{2}^{\;r}|}^{s}+{|\lambda_{1}^{\;r}-\lambda_{2}^{\;r}|}^{s}\right]$

$$=\frac{1}{2}\sum_{j=1}^{n}\left[{|\gamma_{2}^{\;r}-\gamma_{1}^{\;r}|}^{s}+{|\lambda_{2}^{\;r}-\lambda_{1}^{\;r}|}^{s}\right]=D\left(\vartheta_{2},\vartheta_{1}\right)$$

(iv) Finally, consider three FOFNs *ϑ*_1_, *ϑ*_2_, *ϑ*_3_. Since $|\gamma_{1}^{\;r}-\gamma_{3}^{\;r}|\leq |\gamma_{1}^{\;r}-\gamma_{2}^{\;r}|+|\gamma_{2}^{\;r}-\gamma_{3}^{\;r}|$ and 0 < *q*/*p* ≤ 1, we have ${|\gamma_{1}^{\;r}-\gamma_{3}^{\;r}|}^{s}\leq {\left(|\gamma_{1}^{\;r}-\gamma_{2}^{\;r}|+|\gamma_{2}^{\;r}-\gamma_{3}^{\;r}|\right)}^{s}\leq {|\gamma_{1}^{\;r}-\gamma_{2}^{\;r}|}^{s}+{|\gamma_{2}^{\;r}-\gamma_{3}^{\;r}|}^{s}$. Similarly, $\begin{array}{c} {|\lambda_{1}^{\;r}-\lambda_{3}^{\;r}|}^{s}\leq {|\lambda_{1}^{\;r}-\lambda_{2}^{\;r}|}^{s}+{|\lambda_{2}^{\;r}-\lambda_{3}^{\;r}|}^{s}. \end{array}$ Adding the above two inequalities, multiplying by 1/2, gives $D\left(\vartheta_{1},\vartheta_{3}\right)\leq \frac{1}{2}\sum_{j=1}^{n}\left[{|\gamma_{1}^{\;r}-\gamma_{2}^{\;r}|}^{s}+{|\lambda_{1}^{\;r}-\lambda_{2}^{\;r}|}^{s}\right]+\frac{1}{2}\sum_{j=1}^{n}\left[{|\gamma_{2}^{\;r}-\gamma_{3}^{\;r}|}^{s}+{|\lambda_{2}^{\;r}-\lambda_{3}^{\;r}|}^{s}\right]=D\left(\vartheta_{1},\vartheta_{2}\right)+D\left(\vartheta_{2},\vartheta_{3}\right)$. Therefore, 𝒟(*ϑ*_1_, *ϑ*_3_) ≤ 𝒟(*ϑ*_1_, *ϑ*_2_) + 𝒟(*ϑ*_2_, *ϑ*_3_).

Hence, the proposed distance measure satisfies non-negativity, identity of indiscernibles, symmetry, and the triangle inequality. Therefore, 𝒟 is a valid distance measure for *p* ≥ *q* > 0. □

## 4. Proposed MCGDM Framework in the FOF Environment

Consider a multi-criteria group decision-making (MCGDM) problem involving *m* alternatives and *n* evaluation criteria, represented by *Alt* = {*Alt*_1_, *Alt*_2_, …., *Alt_m_*} and *Cri* = {*Cri*_1_, *Cri*_2_, …, *Cri_n_*}, respectively. Let *N* decision makers participate in the evaluation process, denoted by *Exp* = {*Exp*_1_, *Exp*_2_, …, *Exp_N_*}. Each criterion *Cri_j_* is associated with a weight *ϖ_j_*, satisfying $0\leq \varpi_{j}\leq 1,\sum_{j=1}^{n}\varpi_{j}=1.$ The decision-maker weights are represented by *θ* = (*θ*_1_, *θ*_2_, …, *θ_N_*), where 0 ≤ *θ_t_* ≤ 1, $\sum_{t=1}^{N}\theta_{t}=1$. Each decision maker *Exp_t_* evaluates alternative *Alt_i_* with respect to criterion *Cri_j_* using a FOFN $\Lambda_{\mathrm{ij}}^{t}=\left(\gamma_{\mathrm{ij}}^{t},\lambda_{\mathrm{ij}}^{t}\right),$ where *i* = 1, 2, …, *m*, *j* = 1, 2, …, *n*, and *t* = 1, 2, …, *N*. The proposed MCGDM framework consists of the following sequential steps.

Step 1. Construction of the FOFN-Based Decision Matrices: Each decision maker independently provides an evaluation of every alternative under each criterion. Accordingly, for the *t^th^* decision maker, the FOFN-based decision matrix is constructed as $\Lambda^{t}={\left(E_{\mathrm{ij}}^{t}\right)}_{m\times n}={\left\langle \left(\gamma_{\mathrm{ij}}^{t},\lambda_{\mathrm{ij}}^{t}\right)\right\rangle }_{m\times n},t=1,2,\ldots ,N.$ Here, $\vartheta_{\mathrm{ij}}^{t}$ denotes the assessment of alternative *Alt_i_* with respect to criterion *Cri_j_* provided by decision maker *Exp_t_*. Thus,

(9) $$\Lambda^{t}=\left(\begin{array}{cccc} \Lambda_{11}^{t} & \Lambda_{12}^{t} & \ldots & \Lambda_{1n}^{t}\\ \Lambda_{21}^{t} & \Lambda_{22}^{t} & \ldots & \Lambda_{2n}^{t}\\ \vdots & \vdots & \ddots & \vdots \\ \Lambda_{m1}^{t} & \Lambda_{m2}^{t} & \ldots & \Lambda_{\mathrm{mn}}^{t} \end{array}\right)$$

Consequently, *N* individual FOFN-based decision matrices are obtained, one from each decision maker.

Step 2. Determination of Decision-Maker Weights Using AHP: Since the decision makers may have different levels of expertise and relative importance, their weights are determined using the Analytic Hierarchy Process (AHP). Let (*θ*_1_, *θ*_2_, …, *θ_N_*) denote the decision-maker weight vector, where *θ_t_* ≥ 0, $\sum_{t=1}^{N}\theta_{t}=1.$ The determination of these weights consists of the following phases.

Phase 1. Construction of the pairwise comparison matrix: A pairwise comparison matrix $A={\left(a_{\mathrm{st}}\right)}_{N\times N}$ is constructed to represent the relative importance of decision maker *Exp_s_* over decision maker *Exp_t_*. The comparisons are established according to Saaty’s fundamental scale. The matrix satisfies $a_{\mathrm{ss}}=1,a_{\mathrm{ts}}=\frac{1}{a_{\mathrm{st}}},a_{\mathrm{st}}>0.$ Hence,

$$A=\left(\begin{array}{cccc} 1 & a_{12} & \ldots & a_{1N}\\ 1/a_{12} & 1 & \ldots & a_{2N}\\ \vdots & \vdots & \ddots & \vdots \\ 1/a_{1N} & 1/a_{2N} & \ldots & 1 \end{array}\right).$$

Phase 2. Normalization of the comparison matrix: First, calculate the sum of the elements in each column: $S_{t}^{*}=\sum_{s=1}^{N}a_{\mathrm{st}},t=1,2,\ldots ,N.$ Each element of the comparison matrix is then normalized according to ${\tilde{a}}_{\mathrm{st}}=\frac{a_{\mathrm{st}}}{S_{t}^{*}}.$ The normalized comparison matrix is therefore given by $\tilde{A}={\left({\tilde{a}}_{\mathrm{st}}\right)}_{N\times N}.$

Phase 3. Calculation of decision-maker weights: The priority weight associated with decision maker *Exp_s_* is obtained by averaging the elements of the corresponding normalized row $\theta_{s}=\frac{1}{N}\sum_{t=1}^{N}{\tilde{a}}_{\mathrm{st}},s=1,2,\ldots ,N.$ Accordingly, (*θ*_1_, *θ*_2_, …, *θ_N_*) is obtained as the decision-maker weight vector. This vector provides the relative importance of the decision makers in the subsequent aggregation process. The resulting vector approximates the principal eigenvector associated with *Aθ* = *λ*_max_*θ*.

Step 3. Aggregation of Individual Assessments: After determining the decision-maker weights, the individual FOFN assessments are aggregated to obtain a collective assessment for each alternative-criterion pair. The proposed FOFEWA operator is employed for this purpose. For $\vartheta_{\mathrm{ij}}^{1},\vartheta_{\mathrm{ij}}^{2},\ldots ,\vartheta_{\mathrm{ij}}^{N}$, the aggregated assessment is expressed as

(10) $$R_{\mathrm{ij}}=\mathrm{FOFEWA}\left(\vartheta_{\mathrm{ij}}^{1},\vartheta_{\mathrm{ij}}^{2},\ldots ,\vartheta_{\mathrm{ij}}^{N}\right)=\overset{N}{\underset{t=1}{⨁}}\theta_{t}\vartheta_{\mathrm{ij}}^{t}$$

The resulting collective FOFN is obtained from the proposed Einstein-based operational laws as

(11) $$R_{\mathrm{ij}}=\left(\begin{array}{c} {\left(\frac{{\prod_{t=1}^{N}\left(1+{\left(\gamma_{\mathrm{ij}}^{t}\right)}^{p/q}\right)}^{\theta_{t}}-{\prod_{t=1}^{N}\left(1-{\left(\gamma_{\mathrm{ij}}^{t}\right)}^{p/q}\right)}^{\theta_{t}}}{{\prod_{t=1}^{N}\left(1+{\left(\gamma_{\mathrm{ij}}^{t}\right)}^{p/q}\right)}^{\theta_{t}}+{\prod_{t=1}^{N}\left(1-{\left(\gamma_{\mathrm{ij}}^{t}\right)}^{p/q}\right)}^{\theta_{t}}}\right)}^{q/p}\\ ,\frac{{\left(2\right)}^{q/p}{\prod_{t=1}^{N}\left({\left(\lambda_{\mathrm{ij}}^{t}\right)}^{p/q}\right)}^{\theta_{t}}}{{\prod_{t=1}^{N}\left(2-{\left(\lambda_{\mathrm{ij}}^{t}\right)}^{p/q}\right)}^{\theta_{t}}+\prod_{t=1}^{N}\left({\left(\lambda_{\mathrm{ij}}^{t}\right)}^{\theta_{t}}\right)} \end{array}\right)$$

Thus, the individual assessments of all decision makers are synthesized into a single collective FOFN-based decision matrix denoted by $\Lambda ={\left(R_{\mathrm{ij}}\right)}_{m\times n}$.

Step 4. Normalization of the Collective Decision Matrix: The collective decision matrix Λ may contain both benefit and cost criteria. Since these criteria have opposite directions of preference, normalization is required before determining the objective criterion weights.

For each *i* = 1, 2, …, *m* and *j* = 1, 2, …, *n*, the normalized FOFN *r_ij_* is defined by

(12) $$r_{\mathrm{ij}}=\left\{\begin{array}{cc} \left(\gamma_{\mathrm{ij}},\lambda_{\mathrm{ij}}\right), & j\in B,\\ \left(\lambda_{\mathrm{ij}},\gamma_{\mathrm{ij}}\right), & j\in C, \end{array}\right.$$

where *B* and *C* denote the sets of benefit and cost criteria, respectively.

Accordingly, the normalized decision matrix is represented by $\tilde{\Lambda }={\left(r_{\mathrm{ij}}\right)}_{m\times n}.$ This transformation places benefit and cost criteria on a common preference orientation and facilitates their subsequent treatment within the decision-making procedure.

Step 5. Determination of Objective Criterion Weights: To determine the relative importance of the criteria objectively, the extended entropy method is applied to the normalized decision matrix. For each criterion *Cri_j_*, its entropy-based weight is calculated as

(13) $$\varpi_{j}=\frac{1+\sum_{i=1}^{m}\left[\gamma_{\mathrm{ij}}\ln\left(\gamma_{\mathrm{ij}}\right)+\lambda_{\mathrm{ij}}\ln\left(\lambda_{\mathrm{ij}}\right)\right]}{\sum_{j=1}^{n}\left\{1+\sum_{i=1}^{m}\left[\gamma_{\mathrm{ij}}\ln\left(\gamma_{\mathrm{ij}}\right)+\lambda_{\mathrm{ij}}\ln\left(\lambda_{\mathrm{ij}}\right)\right]\right\}}$$

The resulting criterion-weight vector is *ϖ* = (*ϖ*_1_, *ϖ*_2_, …, *ϖ_n_*), with $0\leq \varpi_{j}\leq 1,\sum_{j=1}^{n}\varpi_{j}=1.$ Thus, the entropy procedure assigns larger weights to criteria containing greater discriminating information within the decision matrix.

**Remark** **1.** *The entropy value associated with criterion C_j_ is calculated from the aggregated fractional orthopair fuzzy information using* $e_{j}=1+\sum_{i=1}^{m}\left[\gamma_{\mathrm{ij}}\ln\left(\gamma_{\mathrm{ij}}\right)+\lambda_{\mathrm{ij}}\ln\left(\lambda_{\mathrm{ij}}\right)\right].$ *The convention* 0ln0 = 0 *is adopted based on* $\lim_{x\to 0^{+}}x\ln x=0$*. The entropy values are then normalized according to* $\varpi_{j}=\frac{e_{j}}{\sum_{j=1}^{n}e_{j}},$ *so that* $\sum_{j=1}^{n}\varpi_{j}=1$*.*

Step 6. Construction of the Weighted Aggregated Decision Matrix: The normalized decision matrix is subsequently weighted using the objective criterion weights. For each alternative *Alt_i_* and criterion *Cri_j_*, the weighted FOFN is obtained as *Q_ij_* = *ϖ_j_r_ij_*. Under the proposed operational law (Definition 4), the weighted assessment is expressed as

(14) $$Q_{\mathrm{ij}}=\varpi_{j}r_{\mathrm{ij}}=\left(\begin{array}{c} {\left(\frac{{\left(1+\gamma_{\mathrm{ij}}^{p/q}\right)}^{\varpi_{j}}-{\left(1-\gamma_{\mathrm{ij}}^{p/q}\right)}^{\varpi_{j}}}{{\left(1+\gamma_{\mathrm{ij}}^{p/q}\right)}^{\varpi_{j}}+{\left(1-\gamma_{\mathrm{ij}}^{p/q}\right)}^{\varpi_{j}}}\right)}^{q/p},\\ \frac{{\left(2\right)}^{q/p}{\left(\lambda_{1}\right)}^{\varpi_{j}}}{{\left({\left(2-\lambda_{\mathrm{ij}}^{p/q}\right)}^{\varpi_{j}}+{\left(\lambda_{\mathrm{ij}}^{p/q}\right)}^{\varpi_{j}}\right)}^{q/p}} \end{array}\right)$$

Consequently, the weighted aggregated decision matrix is given by $Q={\left(Q_{\mathrm{ij}}\right)}_{m\times n}$. This matrix incorporates both the normalized performance of the alternatives and the objective importance of the criteria.

Step 7. Determination of Positive and Negative Ideal Solutions: The positive ideal solution (PIS) and negative ideal solution (NIS) are determined from the weighted aggregated decision matrix. For each criterion *Cri_j_*, the PIS is defined as

(15) $$H_{j}^{+}=\left(\gamma_{j}^{+},\lambda_{j}^{+}\right)=\left(\underset{i}{\max}\left(\gamma_{\mathrm{ij}}\right),\underset{i}{\min}\left(\lambda_{\mathrm{ij}}\right)\right),$$

whereas the NIS is given by

(16) $$H_{j}^{-}=\left(\gamma_{j}^{-},\lambda_{j}^{-}\right)=\left(\underset{i}{\min}\left(\gamma_{\mathrm{ij}}\right),\underset{i}{\max}\left(\lambda_{\mathrm{ij}}\right)\right).$$

Thus, $H^{+}=\left(H_{1}^{+},H_{2}^{+},\ldots ,H_{n}^{+}\right)$ represents the most desirable performance profile, while $H^{-}=\left(H_{1}^{-},H_{2}^{-},\ldots ,H_{n}^{-}\right)$ represents the least desirable performance profile. Here, *i* = 1, 2, …, *m*, *j* = 1, 2, …, *n*.

Step 8. Calculation of the Distances from the Ideal Solutions: The relative performance of each alternative is evaluated by calculating its distance from the PIS and NIS. For alternative *Alt_i_*, the distance from the PIS is calculated as

(17) $$D\left(Q_{i},H^{+}\right)=\frac{1}{2}\sum_{j=1}^{n}\left[{|\gamma_{\mathrm{ij}}^{p/q}-{\left(\gamma_{j}^{+}\right)}^{p/q}|}^{q/p}+{|\lambda_{\mathrm{ij}}^{p/q}-{\left(\lambda_{j}^{+}\right)}^{p/q}|}^{q/p}\right].$$

Similarly, the distance from the NIS is determined by

(18) $$D\left(Q_{i},H^{-}\right)=\frac{1}{2}\sum_{j=1}^{n}\left[{|\gamma_{\mathrm{ij}}^{p/q}-{\left(\gamma_{j}^{-}\right)}^{p/q}|}^{q/p}+{|\lambda_{\mathrm{ij}}^{p/q}-{\left(\lambda_{j}^{-}\right)}^{p/q}|}^{q/p}\right].$$

Here, $\left(\gamma_{j}^{+},\lambda_{j}^{+}\right)$ and $\left(\gamma_{j}^{-},\lambda_{j}^{-}\right)$ denote the membership and non-membership components of the PIS and NIS, respectively.

A smaller value of 𝒟(*Q_i_*, *H*^+^) indicates that alternative *Alt_i_* is closer to the most desirable solution, whereas a larger value of 𝒟(*Q_i_*, *H*^−^) indicates that the alternative is farther from the least desirable solution.

Step 9. Calculation of the Closeness Index and Ranking of Alternatives: Finally, the relative desirability of each alternative is determined using the closeness index. For alternative *Alt_i_*, the closeness index is calculated as

(19) $$\Phi_{i}=\frac{D\left(Q_{i},H^{-}\right)}{\left(D\left(Q_{i},H^{-}\right)\right)+\left(D\left(Q_{i},H^{+}\right)\right)}.$$

The alternatives are subsequently ranked according to their corresponding Φ*_i_* values. A higher value of Φ*_i_* indicates a preferable alternative.

**Figure 1:**
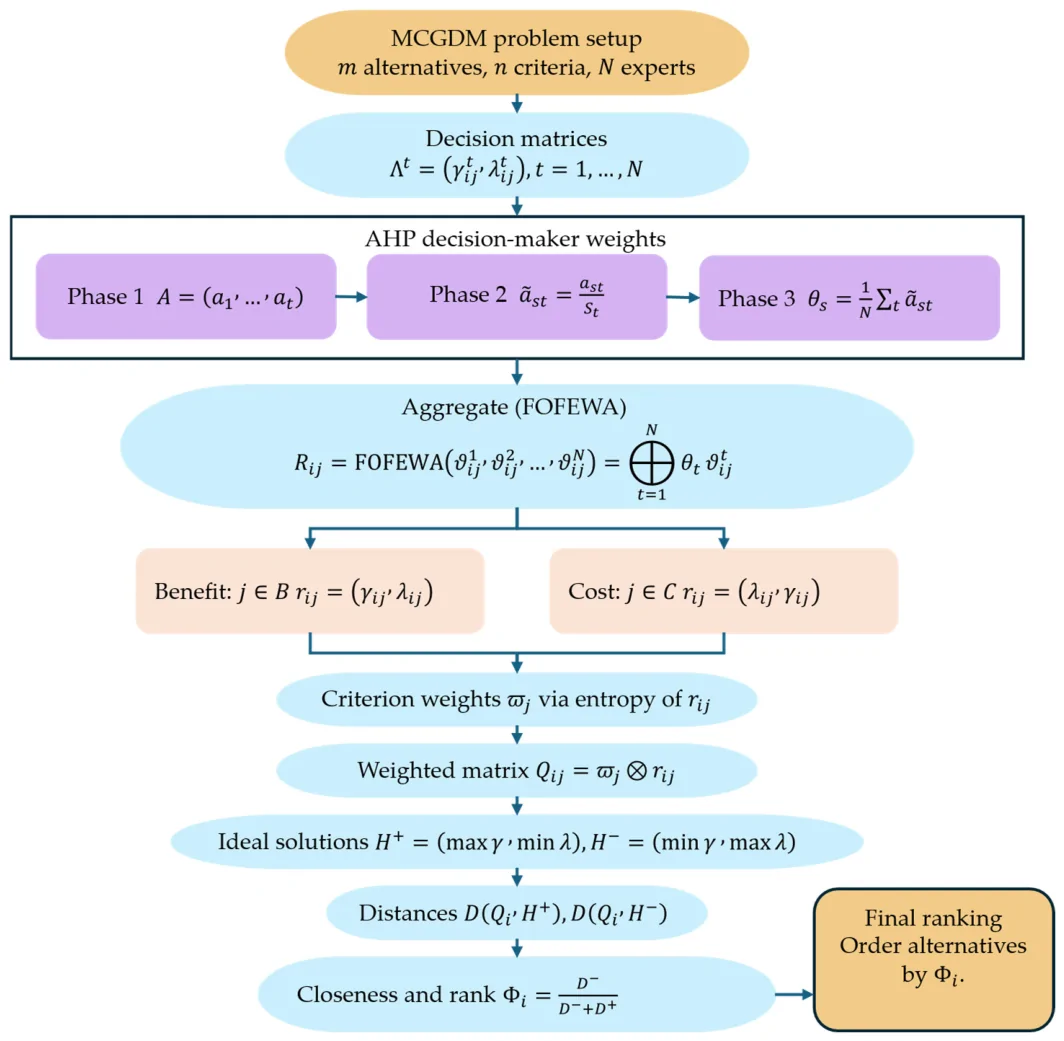
Flowchart of the proposed MCGDM methodology.

The overall computational procedure of the proposed MCGDM approach is illustrated in Figure 1, which presents the sequential steps from problem formulation and expert-weight determination to aggregation, criterion weighting, ideal-solution construction, distance calculation, and final ranking of alternatives.

## 5. Numerical Illustration

In this section, the effectiveness and practical relevance of the developed approach are investigated. To illustrate its applicability, the method is implemented in a numerical case study concerning the prioritization of renewable power generation technologies under conditions of unknown criteria weights.

### 5.1. Case Study

The choice of renewable power generation technology is a strategically important and naturally very challenging decision-making process that simultaneously combines technical feasibility, economic performance, environmental sustainability, operational reliability and policy correspondence. All renewable technologies present different features in terms of resource dependency, capital intensity, level of maturity, potential for integration into the grid and their effects on the long-term system. This has meant that the appraisal and ranking of the renewable energy alternatives cannot be narrowed down to one performance measure but rather, a holistic appraisal across a myriad of and usually incompatible metrics [60,61,62]. The techniques of MCGDM are prevalent in the field of renewable energy planning. Such analyses require an accurate selection of experts, criteria and alternatives to be effective. Random decisions can lead to subjective assessments or unrealistic judgments [63,64,65]. Involvement of various professionals is essential in the decision-making on renewable energy as they are multidisciplinary in nature. All of these allow technical, economic, environmental, and policy approaches to ensure an objective and fair assessment of technologies [66,67]. For the proposed case study, five experts are invited. The comparative details of experts are listed in Table 1.

Similarly, the selection of evaluation criteria should reflect both theoretical relevance and practical significance. Commonly used criteria in renewable energy assessment include technical performance measures, economic indicators (such as the levelized cost of electricity), environmental factors related to emissions and ecological footprint, as well as social and policy considerations. The inclusion of both benefit-type and cost-type criteria enables a more accurate representation of the trade-offs inherent in renewable technology selection and aligns with established sustainable energy evaluation frameworks [68,69,70].

The eight criteria adopted in this study were selected based on their widespread recognition in renewable energy evaluation frameworks: (1) energy generation efficiency (*Cri*_1_)Efficiency and capacity utilization are primary indicators of technological effectiveness and energy yield potential [71,72,73]; (2) dispatchability and grid reliability contribution (*Cri*_2_) the increasing penetration of variable renewables necessitates evaluation of dispatchability and grid support capabilities [74,75]; (3) environmental sustainability (*Cri*_3_) environmental performance, particularly emissions mitigation, is a central objective of renewable adoption [76,77]; (4) technology maturity and operational reliability (*Cri*_4_) technology readiness and reliability strongly influence investment decisions and risk perception [78]; (5) social acceptance and policy compatibility (*Cri*_5_) empirical studies confirm that social and regulatory factors critically affect renewable project implementation [79]; (6) levelized cost of electricity (*Cri*_6_) levelized cost of electricity remains the most widely used economic comparison metric across generation technologies [80]; (7) resource availability and site constraints (*Cri*_7_) renewable technologies are inherently resource-dependent, requiring consideration of geographic feasibility [81,82]; (8) investment risk and implementation complexity (*Cri*_8_) risk and complexity influence financial viability and project scalability [83,84].

**Figure 2:**
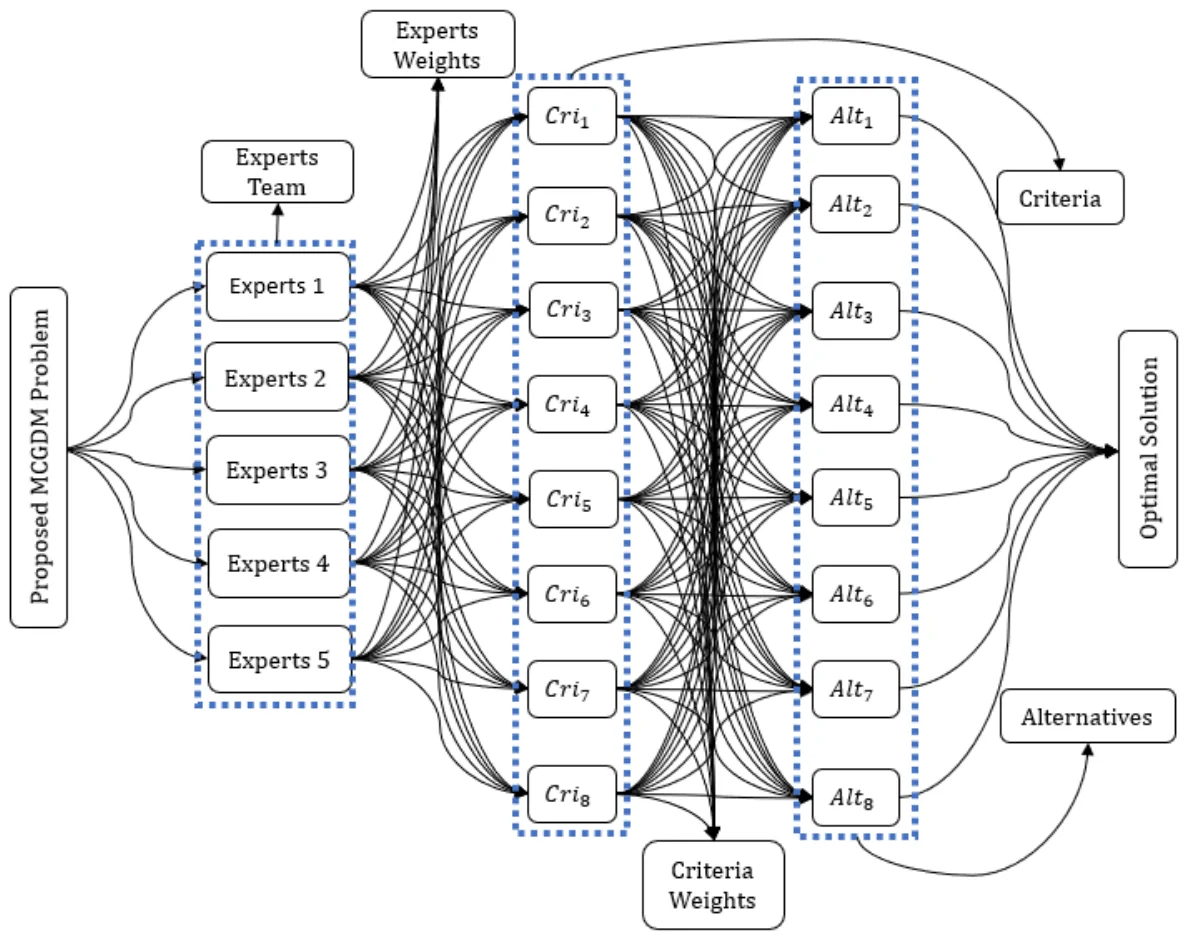
Structure of the proposed MCGDM framework, showing the relationships among experts, evaluation criteria, criteria weights, alternatives, and the resulting optimal solution.

The selection of renewable power options also influences the credibility of the decision model. A representative set of alternatives should include not only mature technologies but also emerging ones, thereby reflecting the ongoing trajectory of technological development and future energy transition trends. Technologies such as solar photovoltaics, wind power, hydropower, biomass, and geothermal energy, along with advanced systems including marine energy and hydrogen-based solutions, are widely discussed in the literature due to their relevance for modern power systems and long-term decarbonization objectives. The chosen alternatives capture diverse technological characteristics and system roles: Solar photovoltaic (*Alt*_1_) and Onshore wind (*Alt*_2_) Globally dominant and economically competitive renewable sources [85,86]. Biomass Power (*Alt*_3_) and Small-scale hydropower (*Alt*_4_) Dispatchable renewables offering reliability advantages [87,88]. Geothermal power (*Alt*_5_) High-capacity factor and base-load capability [89]. Concentrated Solar Power (*Alt*_6_) Storage-enabled solar generation [90]. Wave and tidal energy (*Alt*_7_) Emerging but promising marine technologies [91]. Green hydrogen fuel cell power (*Alt*_8_) Representative of next-generation clean energy systems and storage-coupled solutions [92]. Figure 2 presents the interaction between experts, criteria, alternatives, and weighting mechanisms.

To facilitate expert evaluations and reduce cognitive burden during the decision-making process, qualitative assessments were expressed through a predefined linguistic scale. The experts evaluated each renewable energy alternative under every criterion using nine linguistic terms ranging from very unfavorable to outstanding performance. These linguistic labels were subsequently transformed into Fractional Orthopair Fuzzy Numbers (FOFNs) to enable mathematical aggregation and analysis. The linguistic terms employed in this study are: *L* = {VU, U, SU, N, SF, F, HF, VF, O}, where VU = Very Unfavorable, U = Unfavorable, SU = Slightly Unfavorable, N = Neutral, SF = Slightly Favorable, F = Favorable, HF = Highly Favorable, VF = Very Favorable, O = Outstanding. The corresponding Fractional Orthopair Fuzzy representations are given by: VU = (0.10,0.90), U = (0.20,0.80), SU = (0.35,0.65), N = (0.50,0.50), SF = (0.65,0.35), F = (0.75,0.25), HF = (0.85,0.15), VF = (0.90,0.10), O = (0.95,0.05). Table 2 summarizes the assessments by the five experts in linguistic terms.

The proposed TOPSIS method is applied to solve the renewable technologies evolution problem. The weights of the five experts are determined in the first stage using the AHP method. In the second stage, the criteria weights are calculated using the fractional orthopair method [40]. Finally, the proposed TOPSIS approach is utilized to assess the renewable technology alternatives.

**Stage 1.** In the first stage, the relative importance weights of the five decision experts are determined by employing the standard AHP described in Section 4 (Step 1). The experts include a renewable energy engineer (*Exp*_1_), energy economist (*Exp*_2_), environmental specialist (*Exp*_3_), power system planner (*Exp*_4_), and policy and social expert (*Exp*_5_). Pairwise comparisons were established using reciprocal judgments, where *a_ss_* = 1 and *a_ts_* = 1/*a_st_*. The resulting pairwise comparison matrix is given by:

$$A=\left(\begin{array}{ccccc} 1.00 & 1.10 & 1.20 & 1.20 & 1.40\\ 1/1.10 & 1.00 & 1.10 & 1.10 & 1.20\\ 1/1.20 & 1/1.10 & 1.00 & 1.10 & 1.20\\ 1/1.20 & 1/1.10 & 1/1.10 & 1.00 & 1.10\\ 1/1.40 & 1/1.20 & 1/1.20 & 1/1.10 & 1.00 \end{array}\right).$$

The matrix was normalized column-wise, and the priority vector was obtained by averaging the elements of each normalized row. The resulting expert-weight vector is *w* = (0.2332, 0.2102, 0.1989, 0.1881, 0.1696). The consistency of the pairwise comparison judgments was evaluated using the maximum eigenvalue *λ*_max_, consistency index (CI), and consistency ratio (CR). For the five-expert comparison matrix, *λ*_max_ = 5.0014. Hence, *CI* = 0.00035. For *n* = 5, Saaty’s random index is *RI* = 1.12. Therefore, *CR* = 0.00031. Because *CR* < 0.10, the pairwise comparison matrix satisfies the standard AHP consistency criterion. The resulting expert weights are therefore used in the subsequent aggregation and decision-making calculations.

The evaluations provided by the experts, together with their corresponding weights, are aggregated using the FOFEWA operator as presented in Equation (11) to construct the collective decision matrix, which is summarized in Table 3.

The selected criteria include both cost-type and benefit-type attributes. A detailed description of each criterion is provided in Table 4.

From Table 4, it can be observed that criteria *Cri*_6_ and *Cri*_8_ are cost-type criteria. Hence, normalization of the proposed model is required. Equation (12) was applied to perform the normalization process, and the resulting normalized decision matrix is shown in Table 5.

**Stage 2.** In renewable power generation DM, determining the relative importance of evaluation criteria is a critical step, as it directly influences the prioritization of alternative technologies. In this study, the weights of the evaluation criteria are objectively determined using the entropy method [40], which measures the degree of information provided by each criterion based on the variability of expert evaluations. The criteria weights were computed using Equation (13) and the resulting values are presented in Table 6.

The criteria weights and the aggregated decision matrix are combined using Equation (14) to compute the weighted aggregated values, and the obtained results are summarized in Table 7.

The negative and positive ideal solutions are determined utilizing Equations (15) and (16). The obtained ideal solutions ($H_{j}^{-}$, $H_{j}^{+}$) are: $H_{j}^{-}=\left\{\left(0.015,0.985\right)\right.$, (0.039, 0.961), (0.032, 0.968), (0.027, 0.973), (0.044, 0.956), (0.020, 0.981), (0.030, 0.970), (0.017, 0.983)} and *H*^+^ = {(0.176, 0.824), (0.201,0.799), (0.160, 0.840), (0.147, 0.853), (0.166, 0.835), (0.165, 0.835), (0.158, 0.842), (0.142, 0.858).

The distances of each alternative from negative and positive ideal solutions are computed using Equations (17) and (18). The obtained results are summarized in Table 8.

**Figure 3:**
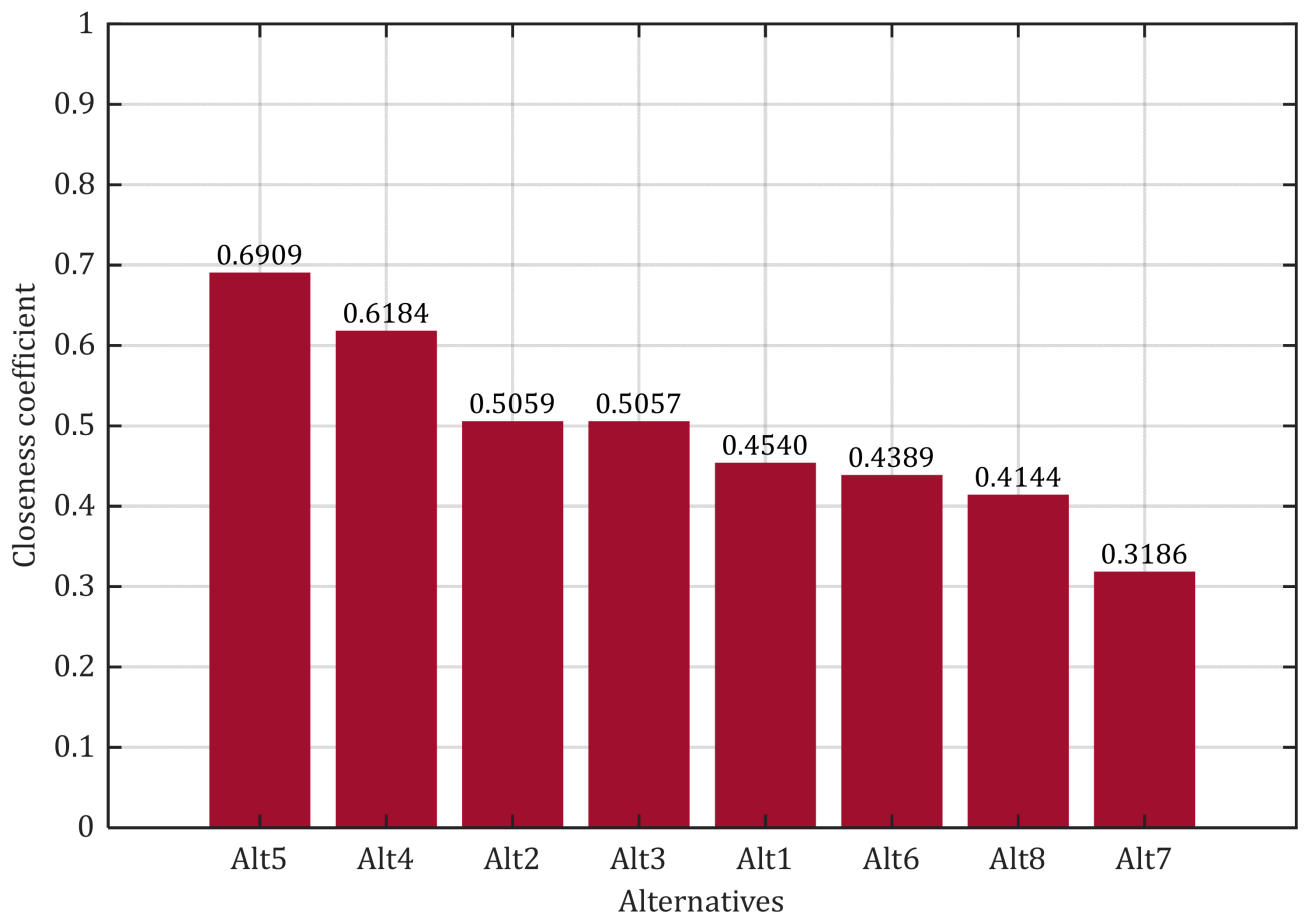
Closeness index values of alternatives, where values nearer to zero indicate better performance and higher ranking.

Equation (19) was used to calculate the revised closeness index Φ for each alternative, and the corresponding results are reported in Table 9. Furthermore, to clearly illustrate the ranking order of the alternatives, the results are graphically presented in Figure 3.

Table 9 presents the relative closeness index (Φ*_i_*) and the corresponding ranking of the eight alternatives considered in the proposed multi-criteria group decision-making (MCGDM) framework for prioritizing renewable power generation technologies. The alternatives were ranked in descending order of Φ*_i_*, where a larger value indicates a closer position to the positive ideal solution and, consequently, better overall performance relative to the other alternatives. *Alt*_5_ obtains the highest closeness index (Φ_5_ = 0.6916) and is therefore ranked first. *Alt*_4_ follows in second place with Φ_4_ = 0.6182. *Alt*_2_ and *Alt*_3_ occupy the third and fourth positions, with closeness indices of 0.5064 and 0.5055, respectively. *Alt*_1_ and *Alt*_6_ are ranked fifth and sixth, with Φ_1_ = 0.4546 and Φ_6_ = 0.4390. *Alt*_8_ is ranked seventh with Φ_8_ = 0.4152, whereas *Alt*_7_ has the lowest closeness index (Φ_7_ = 0.3181) and is ranked eighth. Accordingly, *Alt*_5_ represents the highest-ranked alternative according to the calculated relative closeness values, while *Alt*_7_ shows the lowest relative performance among the evaluated alternatives.

### 5.2. Sensitivity Analysis

A sensitivity analysis was conducted to investigate the influence of the fractional parameters *p* and *q* on the relative performance and ranking of the renewable-power-generation alternatives. Examination of the mathematical structure of the proposed framework shows that *p* and *q* occur through the ratio *r* = *p*/*q*, together with its reciprocal *q*/*p* = 1/*r*, in the fractional orthopair transformations and the associated distance calculations. Consequently, parameter pairs having the same ratio *p*/*q* generate mathematically equivalent parameterizations within the proposed computational pipeline. For example, the pairs (1, 1), (2, 2), (3, 3), and (10, 10) all correspond to *r* = 1, whereas (2, 1), (4, 2), (6, 3), (8, 4), and (10, 5) all correspond to *r* = 2. Therefore, instead of treating every (*p*, *q*) combination as an independent sensitivity case, the analysis is reported in terms of the distinct ratio *r* = *p*/*q*. Under 1 ≤ *q* ≤ *p* ≤ 10, the 55 admissible (*p*, *q*) combinations reduce to 32 distinct values of *r*.

The complete closeness coefficients Φ*_i_* obtained for the 32 distinct values of *r* are reported in Table 10. The table provides a direct assessment of how the relative performance of each alternative changes as the fractional parameter ratio increases. The corresponding ranking patterns extracted from these closeness coefficients are summarized in Table 11.

Table 10 demonstrates that the fractional parameter ratio affects the closeness coefficients in a systematic but alternative-specific manner. The coefficient of *Alt*_5_ decreases from 0.6916 at *r* = 1to 0.5741 at *r* = 10, while *Alt*_4_ decreases from 0.6182 to 0.5605. In contrast, the coefficients of *Alt*_6_, *Alt*_7_, and *Alt*_8_ increase over the same interval, with *Alt*_6_ increasing from 0.4390 to 0.4649, *Alt*_7_ from 0.3181 to 0.3386, and *Alt*_8_ from 0.4152 to 0.4852. *Alt*_1_ initially increases from 0.4546 to 0.4558 and subsequently decreases to 0.4445. These changes demonstrate that the proposed framework is not insensitive to the fractional parameterization, even though the leading and trailing alternatives retain their positions throughout the investigated range.

The corresponding ranking configurations are presented in Table 11. At *r* = 1, the ranking is *Alt*_5_ > *Alt*_4_ > *Alt*_2_ > *Alt*_3_ > *Alt*_1_ > *Alt*_6_ > *Alt*_8_ > *Alt*_7_. When *r* increases above 1, *Alt*_3_ moves ahead of *Alt*_2_, indicating that the relative ordering of these two closely performing alternatives is sensitive to the fractional parameterization. Further changes occur among *Alt*_1_, *Alt*_6_, and *Alt*_8_ as *r* increases. The Alternative *Alt*_6_ overtakes *Alt*_1_ at approximately *r* = 2.50, while *Alt*_8_ subsequently moves ahead of *Alt*_1_ and *Alt*_6_. At larger values of *r*, *Alt*_8_ also moves ahead of *Alt*_3_ and becomes the third-ranked alternative. These rank transitions provide direct evidence that the sensitivity analysis can reveal parameter-dependent changes rather than producing an invariant ranking for all alternatives.

The sensitivity patterns in Table 11 provide a more informative interpretation than simply reporting an unchanged ranking. First, *Alt*_5_ remains the highest-ranked alternative and *Alt*_7_ remains the lowest-ranked alternative throughout the entire investigated range 1 ≤ *r* ≤ 10, while *Alt*_4_ consistently occupies the second position. Thus, the principal identification of the strongest and weakest alternatives is stable with respect to the fractional parameter ratio. Second, the middle positions exhibit meaningful sensitivity. The ranking of *Alt*_2_ and *Alt*_3_ changes immediately after *r* = 1, reflecting their close performance at the baseline. At *r* = 1, their closeness coefficients are 0.5064 and 0.5055, respectively, whereas at *r* = 1.11 they become 0.5022 and 0.5044, resulting in a reversal of their relative positions. This behavior indicates that the parameter ratio has a measurable influence when alternatives have closely separated closeness coefficients.

A further parameter-dependent transition is observed among *Alt*_1_, *Alt*_6_, and *Alt*_8_. *Alt*_1_ has a closeness coefficient of 0.4546 at *r* = 1, compared with 0.4390 and 0.4152 for *Alt*_6_ and *Alt*_8_, respectively. As *r* increases, the coefficient of *Alt*_8_ rises continuously, reaching 0.4516 at *r* = 2.50, 0.4654 at *r* = 4.00, and 0.4852 at *r* = 10. Consequently, *Alt*_8_ moves progressively upward in the ranking and becomes the third-ranked alternative for *r* = 8.00–10.00. Conversely, *Alt*_1_ declines from 0.4546 at *r* = 1 to 0.4445 at *r* = 10, eventually occupying the seventh position. These changes demonstrate that the proposed fractional parameterization has a substantive effect on the relative assessment of alternatives rather than merely changing the numerical scale of the closeness coefficients. The overall dispersion of the closeness coefficients also changes with *r*. At *r* = 1, the difference between the largest and smallest coefficients is 0.6909 − 0.3186 = 0.3723, whereas at *r* = 10 it decreases to 0.5741 − 0.3386 = 0.2355.

**Figure 4:**
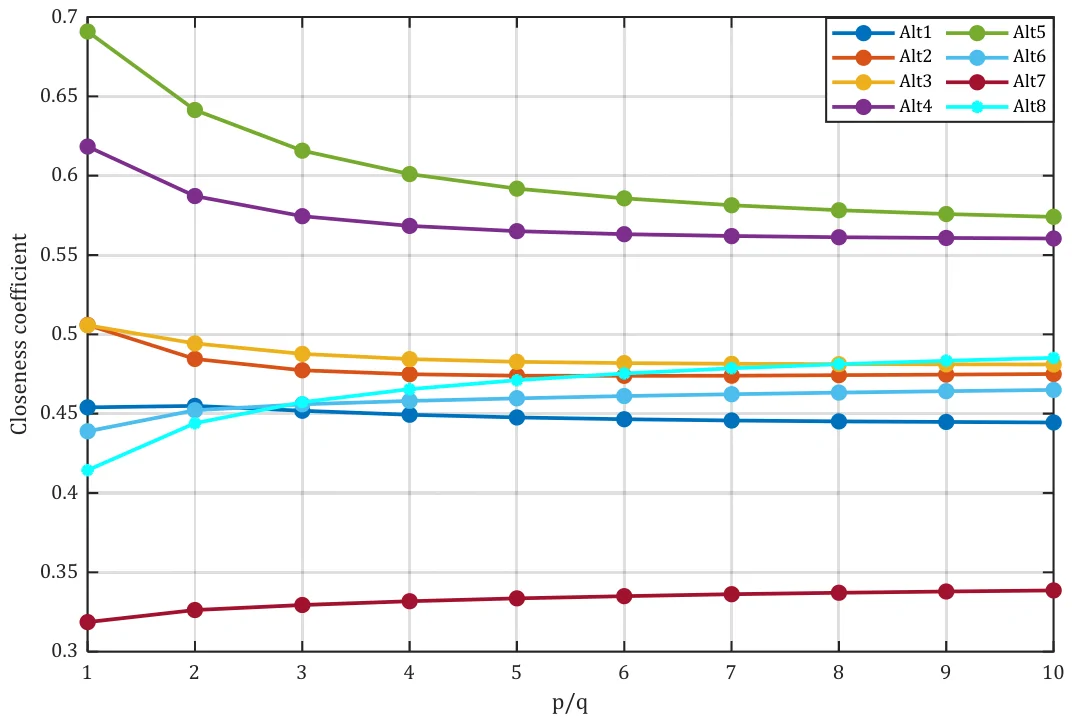
Sensitivity of Φ*_i_* to the fractional-orthopair parameter *r* = *p*/*q*.

Hence, the range of the closeness coefficients contracts as *r* increases. This indicates a progressive reduction in the separation between the highest- and lowest-performing alternatives within the present dataset. Such compression should be interpreted as an empirical feature of the proposed aggregation and distance calculations for this case study rather than as a universal property of all Einstein-type operators or all decision datasets. The results in Table 10 and Table 11 provide two complementary findings. The persistent first and last positions indicate that the identification of *Alt*_5_ and *Alt*_7_ is stable over the investigated fractional-parameter range. At the same time, the changes in the intermediate positions, particularly those involving *Alt*_2_–*Alt*_3_ and *Alt*_1_–*Alt*_6_–*Alt*_8_, demonstrate that the model retains meaningful parameter sensitivity. Therefore, the analysis does not support the interpretation that the numerical case study is completely non-discriminating. Rather, the sensitivity is concentrated among alternatives with relatively close performance, whereas the separation between the leading and trailing alternatives is sufficiently pronounced to prevent changes in their positions over the investigated range. As shown in Figure 4, the relative closeness coefficients vary with the parameter ratio *p*/*q*, while *Alt*_5_ consistently achieves the highest values.

### 5.3. Weight-Wise Sensitivity Analysis

In order to analyze the stability and reliability of the proposed fractional orthopair fuzzy Einstein-TOPSIS, weight-wise sensitivity analysis is carried out. Sensitivity analysis is used to assess the sensitivity of the criteria weight to rank the renewable power generation alternatives. Because renewable energy planning in the real world requires an adjustment in the priorities of the stakeholders and policy preferences, which cannot be predicted with certainty, it is crucial to validate the ranking strength with respect to criteria weight to justify the suggested MCGDM model.

In this analysis, the weight of the most influential criterion, which is levelized cost of electricity (*Cri*_6_) is varied systematically and the weight of the rest of the criteria is varied proportionately to ensure the normalization condition $\sum_{j=1}^{n}\varpi_{j}=1$. The weight of *Cri*_6_ is gradually increased from 0.05 to 0.30, representing decision environments ranging from environmentally driven policies to strongly cost-oriented investment strategies.

The results (Table 12) demonstrate that the ranking structure remains highly stable despite significant variation in the weight of the economic criterion. Alternative *Alt*_5_ consistently retains the highest closeness index across all tested weight configurations, confirming its robustness as the optimal renewable power generation technology. Economically competitive alternatives, e.g., *Alt*_1_, improve in terms of the values of closeness slightly as the importance of the cost criterion increases, at the same time environmentally dominant alternatives improve marginally. But such differences do not change the overall ranking order, which means that the suggested decision framework is not sensitive to moderate weight changes. Moreover, the lesser-ranked options (Alternatives 7 and 8) are kept unchanged during the analysis, which can indicate that the relatively worse performance is organically predetermined, and does not depend on the weight. This pattern proves that the proposed aggregation process is efficient in balancing various dimensions of evaluation without the emergence of ranking of instability.

### 5.4. Comparative Analysis

To assess the consistency of the proposed Einstein-based framework, a comparative analysis was conducted using the same renewable power generation decision problem. The comparison considered the *q*-rung orthopair fuzzy Heronian mean operator [93], cosine-similarity-based decision framework [94], MARCOS [95], hybrid *q*-rung orthopair fuzzy Yager aggregation model [96], two *q*-rung orthopair fuzzy TOPSIS formulations [97,98], evaluation based on distance from average solution (EDAS) [99], weighted distance-based assessment (WDBA) [100], and multi-attributive border approximation area comparison (MABAC) [101]. These methods employ different aggregation, similarity, distance, and ranking mechanisms. The analysis used the same eight alternatives, eight evaluation criteria, five experts, and linguistic assessment information presented in Section 5.

The same linguistic information was used across all considered approaches, as the linguistic terms as defined in Section 5 satisfy the constraints of the respective fuzzy representations. Accordingly, the original linguistic assessments were retained for each method, while the corresponding fuzzy values were constructed according to its mathematical formulation. Each approach was then implemented using its specific aggregation, normalization, distance, similarity, or appraisal procedure. Also, the criterion weights were retained from the entropy-based weighting procedure in Section 5.2 and are given by *ϖ* = (0.1435, 0.1183, 0.1136, 0.0887, 0.1060, 0.1456, 0.1448, 0.1391) where the elements correspond to *Cr*_1_, …, *Cr*_8_, respectively. The expert weights obtained through the AHP procedure were approximately 0.23, 0.21, 0.20, 0.19, and 0.17. These criteria and expert weights were maintained across the comparative experiments wherever applicable, thereby keeping the underlying decision information consistent. For the proposed FOF-Einstein-TOPSIS framework, the baseline setting was *p* = *q* = 1, corresponding to *r* = *p*/*q* = 1. The influence of the fractional parameters was examined separately through the sensitivity analyses in Table 10 and Table 11. For the *q*-rung orthopair fuzzy approaches, *q* = 2 was adopted, while the Heronian mean parameters were set to *a* = *b* = 1. The hybrid Yager model used the parameter specified in [96]. The remaining approaches followed their respective normalization, aggregation, distance, and appraisal procedures.

The comparative calculations were performed using independent Python 3.12 implementations of the proposed and reference methods. The procedures included input transformation, criterion normalization, weighting, method-specific aggregation or distance calculations, and final score generation. For TOPSIS-based methods, the positive and negative ideal solutions were constructed before calculating the corresponding distances and closeness coefficients. For MARCOS, EDAS, WDBA, and MABAC, the relevant reference solutions or appraisal structures were retained according to their respective formulations.

The numerical comparison showed that *Alt*_5_ was identified as the leading alternative by all nine approaches, including the proposed framework, while *Alt*_5_ received the lowest reported score in every method. In the proposed FOF-Einstein-TOPSIS model, *Alt*_5_ obtained a closeness coefficient of Φ_5_ = 0.6916, whereas *Alt*_7_ obtained Φ_7_ = 0.3181, producing a score range of ΔΦ = 0.3735. The *q*-rung orthopair fuzzy Heronian mean method [93] assigned scores of 0.4913 and 0.3712 to *Alt*_5_ and *Alt*_7_, respectively, with ΔΦ = 0.1201. The cosine similarity approach [94] produced scores of 0.4122 for *Alt*_5_ and 0.3683 for *Alt*_7_, resulting in ΔΦ = 0.0439. Under MARCOS [95], the corresponding scores were 0.3214 and 0.2749, with a range of 0.0465. The hybrid Yager model [96] yielded scores of 0.4589 and 0.3571, producing ΔΦ = 0.1018.

For the two *q*-rung orthopair fuzzy TOPSIS formulations, the method in [97] assigned scores of 0.4921 to *Alt*_5_ and 0.3312 to *Alt*_7_, with a score range of 0.1609. The formulation in [98] generated scores of 0.4751 and 0.4157, resulting in ΔΦ = 0.0594. The EDAS method [99] produced scores of 0.5117 and 0.4788, with a range of 0.0329. Similarly, WDBA [100] yielded scores of 0.4249 for *Alt*_5_ and 0.3119 for *Alt*_7_, corresponding to ΔΦ = 0.1130. Finally, MABAC [101] assigned scores of 0.5815 and 0.5170, producing a score range of 0.0645. The key findings of the comparative analysis are summarized in Table 13, which presents the best and worst alternatives, their corresponding scores, and the score range obtained by each method.

The results demonstrate that all nine approaches select *Alt*_5_ as the leading alternative and *Alt*_7_ as the lowest-scoring alternative for the considered decision problem. However, the numerical score ranges vary considerably across the methods, from 0.0329 for EDAS to 0.3735 for the proposed FOF-Einstein-TOPSIS framework. This variation reflects differences in the numerical scales and mathematical operations used by the respective decision-making approaches. Since the score values are generated through different formulations, their absolute magnitudes should not be interpreted as a direct measure of the superiority of one method over another.

The consistent identification of *Alt*_5_ provides evidence of agreement among the evaluated approaches for this decision scenario. Nevertheless, this agreement does not establish the universal superiority of the proposed framework or independently validate the fractional parameterization and Einstein aggregation. The comparative analysis is therefore interpreted as a consistency assessment based on a common assessment structure and weighting information.

The proposed approach was also compared with the existing approach of Zhang et al. [40] under the same parameter settings. The numerical results indicate that the proposed approach is less sensitive to variations in *r* = *p*/*q*. The maximum standard deviation is 0.0378 for the proposed approach, compared with 0.0524 for the existing approach [40], corresponding to a reduction of approximately 27.86%. Similarly, the maximum range decreases from 0.1648 to 0.1175, representing a reduction of approximately 28.70%. The maximum coefficient of variation (CV) is also reduced from 8.41% to 5.93%, corresponding to a reduction of approximately 29.49%. These results indicate that the proposed approach produces smaller variations in the closeness coefficients as *r* changes. The mean Spearman correlation with respect to the baseline ranking at (*r* = 1) is 0.9196, compared with 0.8467 for the existing method [40], representing an improvement of approximately 8.61%. The minimum Spearman coefficient increases from 0.5714 to 0.7143, corresponding to an improvement of approximately 25.01%. Similarly, the mean Kendall’s (*τ*) increases from 0.7143 to 0.8326, representing an improvement of approximately 16.56%, while the minimum value increases from 0.4286 to 0.5714, corresponding to an improvement of approximately 33.36%.

Another important difference is the discriminating ability of the two approaches. The existing method [40] produces tied alternatives at several parameter configurations. For example, at *r* = 1.6, *Alt*_2_ and *Alt*_3_ obtain an identical closeness coefficient of 0.4830 and consequently become tied. Similarly, at *r* = 6, *Alt*_3_ and *Alt*_8_ obtain the same closeness coefficient of 0.4723. In contrast, the proposed approach maintains distinguishable closeness coefficients among the alternatives throughout the investigated configurations. This indicates that the proposed formulation provides greater separation between competing alternatives and reduces the occurrence of ranking ties.

### 5.5. Limitations

The proposed framework has several limitations. First, the procedure involves multiple computational steps, which may increase computational requirements for large-scale decision-making problems. Second, the model depends on the fractional parameters *p* and *q*, and their selection may influence the resulting assessments and rankings. The present sensitivity analysis examines the effect of these parameters while keeping the other inputs fixed; therefore, broader parameter-selection strategies remain a topic for future research. Third, the case study uses illustrative linguistic assessments rather than assessments elicited from recruited experts. Consequently, the numerical results demonstrate the application of the proposed framework under the specified assessment settings rather than providing empirical validation based on real expert responses. In addition, the current model uses fixed expert assessments and does not explicitly incorporate dynamic or behavioral changes in decision environments.

## 6. Conclusions

This study developed a multi-criteria group decision-making framework under the Fractional Orthopair Fuzzy environment for renewable power generation technology prioritization. The framework integrates AHP-based expert weighting, the Fractional Orthopair Fuzzy Einstein Weighted Averaging operator, entropy-based criterion weighting, and distance-based assessment to handle uncertain and heterogeneous evaluation information. Benefit and cost criteria are considered within the proposed decision-making procedure. The framework was applied to a renewable power generation technology selection problem involving technical, economic, environmental, and operational criteria. The resulting calculations provide a ranking of the considered alternatives under the specified assessment information and parameter settings. A sensitivity analysis was conducted by varying the fractional parameters *p* and *q*, while the other inputs were kept fixed. The comparative analysis with existing MCDM approaches was also conducted to examine the consistency of the obtained results. The findings are specific to the assumptions, linguistic assessments, weights, and parameter settings adopted in the case study. Therefore, the results should be interpreted as an application of the proposed framework rather than as evidence of general superiority over existing approaches. The study illustrates how FOF information can be incorporated into an integrated weighting, aggregation, and distance-based decision procedure.

Future research may investigate adaptive parameter-selection mechanisms, elicited expert assessments, alternative weighting schemes, dynamic decision environments, and data-driven techniques for expert and criterion weighting. The framework may also be examined in larger real-world applications, including smart energy systems, healthcare decision-making, and sustainable planning, using additional empirical data to further assess its applicability under different decision-making contexts.

## Figures and Tables

**Table 1:** Functional justification of participating decision experts.

Expert	Primary Role
Renewable Energy Engineer (*Exp*_1_)	Technical feasibility and system performance
Energy Economist (*Exp*_2_)	Cost, lifecycle economics, investment efficiency
Environmental Specialist (*Exp*_3_)	Sustainability and environmental impact
Power System Planner (*Exp*_4_)	Grid integration and reliability
Energy Policy & Social Expert (*Exp*_5_)	Policy alignment and social acceptance

**Table 2:** Assessments provided by five experts.

*Exp*_1_	***Alt_i_***	***Cri*_1_**	***Cri*_2_**	***Cri*_3_**	***Cri*_4_**	***Cri*_5_**	***Cri*_6_**	***Cri*_7_**	***Cri*_8_**
	*Alt*_1_	HF	SF	SU	VF	HF	F	SF	HF
	*Alt*_2_	VF	F	SU	F	VF	VF	SF	F
	*Alt*_3_	SU	SF	F	SU	F	SF	N	SU
	*Alt*_4_	SF	SF	F	VF	F	SU	F	SF
	*Alt*_5_	U	HF	HF	F	F	U	F	SF
	*Alt*_6_	SU	F	SF	VF	SF	SU	SU	SF
	*Alt*_7_	U	SU	SU	VF	SU	U	U	N
	*Alt*_8_	U	N	HF	F	SU	SU	SU	N
*Exp*_2_	*Alt*_1_	VF	N	U	HF	VF	HF	SU	VF
	*Alt*_2_	F	VF	SU	F	HF	F	N	SF
	*Alt*_3_	N	F	VF	U	VF	SU	SF	U
	*Alt*_4_	SF	SF	HF	VF	HF	SU	F	F
	*Alt*_5_	SU	O	HF	F	F	VU	F	SF
	*Alt*_6_	U	HF	F	HF	SF	SU	SU	SF
	*Alt*_7_	VU	SU	U	VF	SU	VU	U	SU
	*Alt*_8_	SU	N	VF	SF	SU	U	SF	N
*Exp*_3_	*Alt*_1_	HF	SF	SU	O	HF	F	N	VF
	*Alt*_2_	F	HF	U	VF	VF	HF	SF	F
	*Alt*_3_	SU	SF	F	U	F	N	SU	U
	*Alt*_4_	N	SF	VF	HF	F	SU	HF	SF
	*Alt*_5_	U	VF	HF	F	HF	VU	F	SF
	*Alt*_6_	SU	F	N	HF	SF	SU	SU	N
	*Alt*_7_	VU	U	SU	VF	SU	VU	U	SU
	*Alt*_8_	U	N	VF	SF	SU	SU	SF	N
*Exp*_4_	*Alt*_1_	VF	N	VU	HF	HF	HF	SU	VF
	*Alt*_2_	SF	VF	SU	F	HF	F	N	SF
	*Alt*_3_	N	SF	HF	SU	VF	SU	SF	U
	*Alt*_4_	SF	N	VF	VF	HF	SU	F	F
	*Alt*_5_	U	O	O	F	F	VU	HF	SF
	*Alt*_6_	SU	HF	SF	HF	SF	SU	SU	SF
	*Alt*_7_	VU	U	SU	HF	SU	VU	U	SU
	*Alt*_8_	SU	SU	VF	SF	N	U	SF	N
*Exp*_5_	*Alt*_1_	O	SF	SU	HF	HF	F	SF	HF
	*Alt*_2_	VF	HF	U	VF	VF	VF	N	F
	*Alt*_3_	SU	SF	VF	U	F	SU	SF	U
	*Alt*_4_	SF	N	HF	O	F	SU	HF	SF
	*Alt*_5_	U	VF	HF	F	HF	VU	F	SF
	*Alt*_6_	U	HF	F	HF	SF	SU	SU	SF
	*Alt*_7_	VU	SU	U	VF	SU	VU	U	SU
	*Alt*_8_	SU	N	VF	SF	SU	U	SF	F

**Table 3:** Aggregated decision matrix.

*Alt_i_*	*Cri*_1_	*Cri*_2_	*Cri*_3_	*Cri*_4_
*Alt*_1_	(0.894,0.106)	(0.595,0.405)	(0.274,0.726)	(0.890,0.110)
*Alt*_2_	(0.813,0.187)	(0.856,0.144)	(0.296,0.704)	(0.820,0.180)
*Alt*_3_	(0.413,0.587)	(0.673,0.327)	(0.839,0.161)	(0.265,0.735)
*Alt*_4_	(0.623,0.377)	(0.601,0.400)	(0.855,0.145)	(0.903,0.097)
*Alt*_5_	(0.233,0.767)	(0.916,0.084)	(0.878,0.122)	(0.750,0.250)
*Alt*_6_	(0.295,0.705)	(0.813,0.188)	(0.667,0.333)	(0.863,0.137)
*Alt*_7_	(0.123,0.877)	(0.293,0.707)	(0.295,0.705)	(0.892,0.108)
*Alt*_8_	(0.287,0.713)	(0.473,0.527)	(0.890,0.110)	(0.676,0.325)
*Alt_i_*	*Cri*_5_	*Cri*_6_	*Cri*_7_	*Cri*_8_
*Alt*_1_	(0.862,0.138)	(0.796,0.205)	(0.513,0.488)	(0.882,0.118)
*Alt*_2_	(0.882,0.118)	(0.842,0.158)	(0.569,0.431)	(0.713,0.287)
*Alt*_3_	(0.825,0.175)	(0.459,0.541)	(0.566,0.434)	(0.236,0.764)
*Alt*_4_	(0.796,0.205)	(0.350,0.650)	(0.792,0.208)	(0.693,0.307)
*Alt*_5_	(0.792,0.208)	(0.123,0.877)	(0.773,0.227)	(0.650,0.350)
*Alt*_6_	(0.650,0.350)	(0.350,0.650)	(0.350,0.650)	(0.623,0.377)
*Alt*_7_	(0.350,0.650)	(0.123,0.877)	(0.200,0.800)	(0.387,0.614)
*Alt*_8_	(0.380,0.620)	(0.266,0.734)	(0.592,0.408)	(0.552,0.448)

**Table 4:** Categorization of criteria.

Criterion	Type	Reason
*Cri*_1_	Benefit	Higher efficiency increases energy output and performance.
*Cri*_2_	Benefit	Greater reliability and grid support are desirable.
*Cri*_3_	Benefit	Better environmental performance and lower emissions are preferred.
*Cri*_4_	Benefit	Mature and reliable technologies reduce uncertainty and failures.
*Cri*_5_	Benefit	Higher acceptance improves implementation success.
*Cri*_6_	Cost	Lower electricity cost is economically preferable.
*Cri*_7_	Benefit	Greater resource availability increases feasibility.
*Cri*_8_	Cost	Lower risk and complexity are desirable.

**Table 5:** Normalized decision matrix.

*Alt_i_*	*Cri*_1_	*Cri*_2_	*Cri*_3_	*Cri*_4_
*Alt*_1_	(0.894,0.106)	(0.595,0.405)	(0.274,0.726)	(0.890,0.110)
*Alt*_2_	(0.813,0.187)	(0.856,0.144)	(0.296,0.704)	(0.820,0.180)
*Alt*_3_	(0.413,0.587)	(0.673,0.327)	(0.839,0.161)	(0.265,0.735)
*Alt*_4_	(0.623,0.377)	(0.601,0.400)	(0.855,0.145)	(0.903,0.097)
*Alt*_5_	(0.233,0.767)	(0.916,0.084)	(0.878,0.122)	(0.750,0.250)
*Alt*_6_	(0.295,0.705)	(0.813,0.188)	(0.667,0.333)	(0.863,0.137)
*Alt*_7_	(0.123,0.877)	(0.293,0.707)	(0.295,0.705)	(0.892,0.108)
*Alt*_8_	(0.287,0.713)	(0.473,0.527)	(0.890,0.110)	(0.676,0.325)
*Alt_i_*	*Cri*_5_	*Cri*_6_	*Cri*_7_	*Cri*_8_
*Alt*_1_	(0.862,0.138)	(0.205,0.796)	(0.513,0.488)	(0.118,0.882)
*Alt*_2_	(0.882,0.118)	(0.158,0.842)	(0.569,0.431)	(0.287,0.713)
*Alt*_3_	(0.825,0.175)	(0.541,0.459)	(0.566,0.434)	(0.764,0.236)
*Alt*_4_	(0.796,0.205)	(0.650,0.350)	(0.792,0.208)	(0.307,0.693)
*Alt*_5_	(0.792,0.208)	(0.877,0.123)	(0.773,0.227)	(0.350,0.650)
*Alt*_6_	(0.650,0.350)	(0.650,0.350)	(0.350,0.650)	(0.377,0.623)
*Alt*_7_	(0.350,0.650)	(0.877,0.123)	(0.200,0.800)	(0.614,0.387)
*Alt*_8_	(0.380,0.620)	(0.734,0.266)	(0.592,0.408)	(0.448,0.552)

**Table 6:** Criteria and their corresponding weights.

Criterion	$1+\sum_{i}\left(\gamma \ln\gamma +\lambda \ln\lambda \right).$	Weight *ϖ_j_*
*Cri*_1_	−3.2822	0.1235
*Cri*_1_	−3.4592	0.1301
*Cri*_1_	−3.0102	0.1133
*Cri*_1_	−2.6487	0.0997
*Cri*_1_	−3.2047	0.1206
*Cri*_1_	−3.2525	0.1224
*Cri*_1_	−3.9314	0.1479
*Cri*_1_	−3.7901	0.1426
Sum		1.0000

**Table 7:** Weighted aggregated decision matrix.

*Alt_i_*	*Cri*_1_	*Cri*_2_	*Cri*_3_	*Cri*_4_
*Alt*_1_	(0.176,0.824)	(0.089,0.911)	(0.032,0.968)	(0.141,0.859)
*Alt*_2_	(0.139,0.861)	(0.165,0.835)	(0.035,0.965)	(0.115,0.885)
*Alt*_3_	(0.054,0.946)	(0.106,0.894)	(0.137,0.863)	(0.027,0.973)
*Alt*_4_	(0.090,0.910)	(0.090,0.910)	(0.143,0.857)	(0.147,0.853)
*Alt*_5_	(0.029,0.971)	(0.201,0.799)	(0.154,0.846)	(0.097,0.903)
*Alt*_6_	(0.038,0.963)	(0.147,0.854)	(0.091,0.909)	(0.129,0.871)
*Alt*_7_	(0.015,0.985)	(0.039,0.961)	(0.034,0.966)	(0.142,0.858)
*Alt*_8_	(0.037,0.964)	(0.067,0.933)	(0.160,0.840)	(0.082,0.918)
*Alt_i_*	*Cri*_5_	*Cri*_6_	*Cri*_7_	*Cri*_8_
*Alt*_1_	(0.156,0.844)	(0.025,0.975)	(0.084,0.917)	(0.017,0.983)
*Alt*_2_	(0.166,0.835)	(0.020,0.981)	(0.095,0.905)	(0.042,0.958)
*Alt*_3_	(0.140,0.860)	(0.074,0.926)	(0.095,0.905)	(0.142,0.858)
*Alt*_4_	(0.130,0.870)	(0.095,0.905)	(0.158,0.842)	(0.045,0.955)
*Alt*_5_	(0.129,0.871)	(0.165,0.835)	(0.151,0.849)	(0.052,0.948)
*Alt*_6_	(0.093,0.907)	(0.095,0.905)	(0.054,0.946)	(0.057,0.944)
*Alt*_7_	(0.044,0.956)	(0.165,0.835)	(0.030,0.970)	(0.102,0.899)
*Alt*_8_	(0.048,0.952)	(0.114,0.886)	(0.100,0.900)	(0.069,0.931)

**Table 8:** Distances between Positive and Negative ideal solutions.

*Alt_i_*	𝓓(𝓠*_i_*, *λ*^+^)	𝓓(𝓠*_i_*, *λ*^−^)
*Alt*_1_	0.5950	0.4960
*Alt*_2_	0.5385	0.5525
*Alt*_3_	0.5395	0.5515
*Alt*_4_	0.4165	0.6745
*Alt*_5_	0.3365	0.7545
*Alt*_6_	0.6120	0.4790
*Alt*_7_	0.7440	0.3470
*Alt*_8_	0.6380	0.4530

**Table 9:** The relative closeness and corresponding ranking order of alternatives.

*Alt_i_*	Φ*_i_*	Ranking
*Alt*_1_	0.4546	5
*Alt*_2_	0.5064	3
*Alt*_3_	0.5055	4
*Alt*_4_	0.6182	2
*Alt*_5_	0.6916	1
*Alt*_6_	0.4390	6
*Alt*_7_	0.3181	8
*Alt*_8_	0.4152	7

**Table 10:** Closeness coefficients Φ*_i_* of the alternatives for the distinct fractional-parameter ratios *r* = *p*/*q*.

*r* = *p*/*q*	*Alt*_1_	*Alt*_2_	*Alt*_3_	*Alt*_4_	*Alt*_5_	*Alt*_6_	*Alt*_7_	*Alt*_8_
1.00	0.4546	0.5064	0.5055	0.6182	0.6916	0.4390	0.3181	0.4152
1.11	0.4548	0.5022	0.5044	0.6130	0.6835	0.4418	0.3202	0.4195
1.13	0.4549	0.5017	0.5042	0.6124	0.6826	0.4421	0.3204	0.4201
1.14	0.4550	0.5012	0.5040	0.6116	0.6815	0.4425	0.3206	0.4208
1.17	0.4551	0.5005	0.5037	0.6106	0.6800	0.4430	0.3209	0.4218
1.20	0.4552	0.4995	0.5033	0.6092	0.6780	0.4437	0.3212	0.4231
1.25	0.4554	0.4981	0.5027	0.6072	0.6751	0.4446	0.3217	0.4249
1.29	0.4555	0.4971	0.5022	0.6059	0.6730	0.4452	0.3221	0.4262
1.33	0.4556	0.4959	0.5016	0.6042	0.6704	0.4460	0.3225	0.4278
1.40	0.4557	0.4944	0.5008	0.6019	0.6668	0.4469	0.3230	0.4299
1.43	0.4558	0.4937	0.5005	0.6010	0.6654	0.4473	0.3232	0.4308
1.50	0.4558	0.4922	0.4996	0.5988	0.6618	0.4482	0.3237	0.4328
1.60	0.4557	0.4903	0.4984	0.5961	0.6572	0.4492	0.3243	0.4355
1.67	0.4557	0.4891	0.4977	0.5943	0.6542	0.4498	0.3247	0.4371
1.75	0.4555	0.4878	0.4968	0.5923	0.6507	0.4505	0.3251	0.4390
1.80	0.4554	0.4871	0.4963	0.5912	0.6487	0.4509	0.3254	0.4401
2.00	0.4549	0.4845	0.4943	0.5872	0.6414	0.4522	0.3262	0.4440
2.25	0.4542	0.4820	0.4922	0.5830	0.6334	0.4534	0.3272	0.4481
2.33	0.4539	0.4813	0.4916	0.5818	0.6311	0.4538	0.3275	0.4493
2.50	0.4534	0.4800	0.4904	0.5796	0.6266	0.4544	0.3280	0.4516
2.67	0.4529	0.4790	0.4894	0.5777	0.6227	0.4549	0.3285	0.4536
3.00	0.4518	0.4774	0.4877	0.5745	0.6158	0.4559	0.3294	0.4573
3.33	0.4509	0.4763	0.4863	0.5720	0.6100	0.4567	0.3303	0.4603
3.50	0.4505	0.4758	0.4858	0.5709	0.6075	0.4571	0.3307	0.4617
4.00	0.4494	0.4748	0.4844	0.5684	0.6011	0.4581	0.3318	0.4654
4.50	0.4484	0.4743	0.4834	0.5665	0.5960	0.4589	0.3327	0.4685
5.00	0.4476	0.4740	0.4827	0.5650	0.5919	0.4597	0.3336	0.4711
6.00	0.4465	0.4738	0.4819	0.5631	0.5857	0.4611	0.3350	0.4753
7.00	0.4457	0.4740	0.4814	0.5619	0.5814	0.4623	0.3362	0.4786
8.00	0.4452	0.4743	0.4812	0.5612	0.5783	0.4633	0.3372	0.4813
9.00	0.4448	0.4746	0.4811	0.5608	0.5759	0.4642	0.3380	0.4834
10.00	0.4445	0.4750	0.4810	0.5605	0.5741	0.4649	0.3386	0.4852

**Table 11:** Ranking order of the renewable-power-generation alternatives across the investigated fractional-parameter ratio *r* = *p*/*q*.

*r* = *p*/*q*	1st	2nd	3rd	4th	5th	6th	7th	8th
1.00	*Alt*_5_	*Alt*_4_	*Alt*_2_	*Alt*_3_	*Alt*_1_	*Alt*_6_	*Alt*_8_	*Alt*_7_
1.11–2.33	*Alt*_5_	*Alt*_4_	*Alt*_3_	*Alt*_2_	*Alt*_1_	*Alt*_6_	*Alt*_8_	*Alt*_7_
2.50	*Alt*_5_	*Alt*_4_	*Alt*_3_	*Alt*_2_	*Alt*_6_	*Alt*_1_	*Alt*_8_	*Alt*_7_
2.67	*Alt*_5_	*Alt*_4_	*Alt*_3_	*Alt*_2_	*Alt*_6_	*Alt*_8_	*Alt*_1_	*Alt*_7_
3.00	*Alt*_5_	*Alt*_4_	*Alt*_3_	*Alt*_2_	*Alt*_8_	*Alt*_6_	*Alt*_1_	*Alt*_7_
3.33–5.00	*Alt*_5_	*Alt*_4_	*Alt*_3_	*Alt*_2_	*Alt*_8_	*Alt*_6_	*Alt*_1_	*Alt*_7_
6.00–7.00	*Alt*_5_	*Alt*_4_	*Alt*_3_	*Alt*_8_	*Alt*_2_	*Alt*_6_	*Alt*_1_	*Alt*_7_
8.00–10.00	*Alt*_5_	*Alt*_4_	*Alt*_8_	*Alt*_3_	*Alt*_2_	*Alt*_6_	*Alt*_1_	*Alt*_7_

**Table 12:** Sensitivity results with respect to weight variation of *Cri*_6_.

Weight of *Cri*_6_	Ranking Order	Best Alternative
0.05	*Alt*_5_ > *Alt*_4_ > *Alt*_2_ > *Alt*_3_ > *Alt*_1_ > *Alt*_6_ > *Alt*_8_ > *Alt*_7_	*Alt*_5_
0.10	*Alt*_5_ > *Alt*_4_ > *Alt*_2_ > *Alt*_3_ > *Alt*_1_ > *Alt*_6_ > *Alt*_8_ > *Alt*_7_	*Alt*_5_
0.15	*Alt*_5_ > *Alt*_4_ > *Alt*_2_ > *Alt*_3_ > *Alt*_1_ > *Alt*_6_ > *Alt*_8_ > *Alt*_7_	*Alt*_5_
0.20	*Alt*_5_ > *Alt*_4_ > *Alt*_2_ > *Alt*_3_ > *Alt*_1_ > *Alt*_6_ > *Alt*_8_ > *Alt*_7_	*Alt*_5_
0.25	*Alt*_5_ > *Alt*_4_ > *Alt*_2_ > *Alt*_3_ > *Alt*_1_ > *Alt*_6_ > *Alt*_8_ > *Alt*_7_	*Alt*_5_
0.30	*Alt*_5_ > *Alt*_4_ > *Alt*_2_ > *Alt*_3_ > *Alt*_1_ > *Alt*_6_ > *Alt*_8_ > *Alt*_7_	*Alt*_5_

**Table 13:** Key findings of the comparative analysis.

Method	Best Alternative (Score)	Worst Alternative (Score)	ΔΦ
Proposed FOF-Einstein-TOPSIS	*Alt*_5_ (0.6916)	*Alt*_7_ (0.3181)	0.3735
*q*-ROF Heronian mean [93]	*Alt*_5_ (0.4913)	*Alt*_7_ (0.3712)	0.1201
Cosine similarity [94]	*Alt*_5_ (0.4122)	*Alt*_7_ (0.3683)	0.0439
MARCOS [95]	*Alt*_5_ (0.3214)	*Alt*_7_ (0.2749)	0.0465
Hybrid Yager [96]	*Alt*_5_ (0.4589)	*Alt*_7_ (0.3571)	0.1018
*q*-ROF TOPSIS [97]	*Alt*_5_ (0.4921)	*Alt*_7_ (0.3312)	0.1609
Gereralized TOPSIS [98]	*Alt*_5_ (0.4751)	*Alt*_7_ (0.4157)	0.0594
EDAS [99]	*Alt*_5_ (0.5117)	*Alt*_7_ (0.4788)	0.0329
WDBA [100]	*Alt*_5_ (0.4249)	*Alt*_7_ (0.3119)	0.1130
MABAC [101]	*Alt*_5_ (0.5815)	*Alt*_7_ (0.5170)	0.0645

## Data Availability

The original contributions presented in this study are included in the article. Further inquiries can be directed to the corresponding authors.
